# Chimeric IL‐6/4R‐LL37 Engineered Macrophages Achieve Synchronized Inflammation Control and Antimicrobial Defence in Sepsis

**DOI:** 10.1002/advs.77016

**Published:** 2026-08-07

**Authors:** Tianyang Jie, Zheyu Zhang, Hao Zhang, Linke Bian, Penglong Zheng, Sijia Zhou, Minqi Wang, Mingyu Qu, Yufei Wang, Xinhua Qu, Bing Yue

**Affiliations:** ^1^ Department of Bone and Joint Surgery Department of Orthopedics Renji Hospital Shanghai Jiaotong University School of Medicine Shanghai China; ^2^ Department of Interventional Oncology Renji Hospital Shanghai Jiao Tong University School of Medicine Shanghai China

**Keywords:** engineered macrophages, immune modulation, in situ transfection, mRNA delivery, sepsis

## Abstract

Treatment of septic patients is confronted with complex immune dysregulation: excessive pro‐inflammatory responses trigger cytokine storm leading to organ damage and high mortality, while concurrent and persistent immune dysfunction manifesting as T cell exhaustion, monocyte and macrophage functional suppression, and impaired antigen presentation significantly increases the risk of secondary infections. Current clinical therapeutic strategies can effectively control infection and cytokine storm, but fail to resolve persistent T cell exhaustion, which is a critical determinant of poor long‐term survival in sepsis patients. This study reported a multifunctional engineered macrophage therapeutic strategy based on in situ LNP delivery of IL‐6/4 fusion protein and LL37 antimicrobial peptide mRNA. The IL‐6/4 fusion blocks IL‐6 mediated pro‐inflammatory signaling and activates IL‐4 mediated anti‐inflammatory pathways, reducing inflammatory injury and promoting tissue repair. LL37 antimicrobial peptide disrupts bacterial membranes to reduce bacterial burden while modulating immune responses. In sepsis mouse models, engineered macrophages significantly improved survival rates, reduced histopathological damage and organ bacterial burden, and effectively restored T cell and macrophage functions, achieving infection control, inflammation modulation, and immune reconstitution. These findings demonstrate the substantial clinical translational potential of this engineered macrophage approach for precision sepsis treatment.

## Graphical abstract

Engineered macrophages programmed in situ by LNP‐delivered IL‐6/4 fusion and LL37 mRNAs simultaneously dampen cytokine storm, promote M2‐like repair, and enhance direct bacterial killing in sepsis. This combinatorial strategy restores T cell and macrophage function, lowers organ bacterial burden, and improves survival, highlighting a precision, host directed cell therapy for difficult to treat infections.

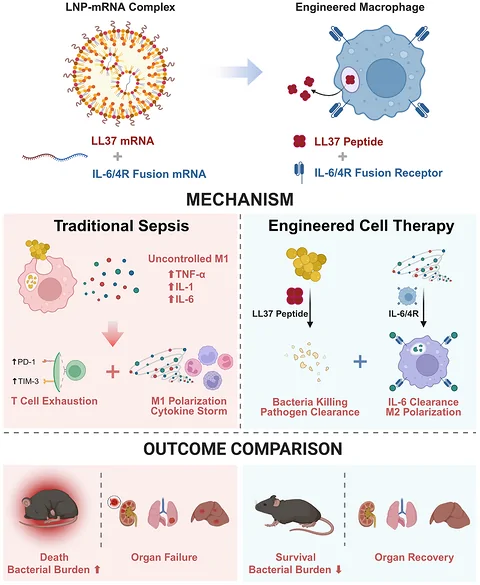

## 1 Introduction

Sepsis is one of the most devastating clinical diseases globally, with an estimated annual incidence of 48.9 million cases worldwide, of which 11 million are fatal, accounting for approximately one‐fifth of global mortality [1]. The core pathological mechanism driving sepsis mortality is the uncontrolled cytokine storm, a hyperinflammatory cascade initiated by pathogen associated molecular patterns (PAMPs) and damage associated molecular patterns (DAMPs) triggering excessive activation of innate immune cells, particularly macrophages, neutrophils, and monocytes [2, 3]. This dysregulated immune activation results in massive release of pro‐inflammatory cytokines including interleukin‐6 (IL‐6), tumor necrosis factor‐α (TNF‐α), and interleukin‐1β (IL‐1β), with IL‐6 levels exceeding 1000 pg/mL in septic patients, over 100‐fold higher than physiological concentrations [4]. Importantly, in sepsis the dysregulation is not limited to cytokine abundance; rather, it is accompanied by myeloid functional reprogramming, where macrophages and monocytes shift toward impaired phagocytosis and aberrant inflammatory transcriptional programs, thereby linking early hyperinflammation to subsequent immune paralysis and persistent organ injury [2, 5]. The cytokine storm participates in three catastrophic consequences: first, direct multi‐organ damage through endothelial dysfunction, microvascular thrombosis, and tissue hypoxia, manifesting as acute respiratory distress syndrome (ARDS), acute kidney injury (AKI), and hepatic failure [6]; second, sustained systemic inflammation driving T cell dysfunction characterized by elevated exhaustion markers (PD‐1, TIM‐3) and functional paralysis [7]; third, compensatory anti‐inflammatory responses transition patients into prolonged immunosuppression phases where impaired pathogen clearance capacity increases susceptibility to secondary infections [5]. This biphasic immune dysregulation, initial hyperinflammation followed by profound immune paralysis, represents the fundamental pathophysiological challenge in sepsis. Currently, only supportive care and antibiotics constitute standard therapeutic approaches, with no specific therapeutics capable of simultaneously controlling cytokine storm mediated tissue damage while preserving antimicrobial defense and preventing immune exhaustion. Because the transition between hyperinflammatory and immunosuppressive phases occurs rapidly and varies across patients, effective therapy must not only dampen pathological inflammation but also preserve (or restore) host defense and immune coordination within a narrow temporal window [5, 7].

Traditional sepsis management relies primarily on broad‐spectrum antibiotics and hemodynamic support to control infection and maintain organ perfusion [8]. However, antimicrobial strategies are fundamentally designed to eradicate pathogens rather than to suppress inflammation, leaving the hyperinflammatory cascade, the primary driver of organ injury, unaddressed [9]. Recent attempts at targeted anti‐inflammatory therapies have exposed a critical therapeutic paradox: interventions that successfully suppress inflammation invariably increase susceptibility to opportunistic infections, while strategies aimed at controlling cytokine storm often fail to achieve effective inflammatory control. Anti‐cytokine monoclonal antibodies, including anti‐TNF‐α (afelimomab) and IL‐1 receptor antagonists (anakinra), have repeatedly failed in sepsis trials due to significantly increased risks of secondary infections [10, 11]. Immune checkpoint inhibitors (anti‐PD‐1 antibodies) theoretically restore T cell function [12] but carry substantial risks of triggering rebound hyperinflammation and cytokine release syndrome [13]. Extracorporeal cytokine removal strategies (hemadsorption devices, high volume hemofiltration) non‐selectively deplete both pro‐ and anti‐inflammatory mediators, disrupting immune homeostasis without improving survival [14]. The fundamental limitation of current immunomodulatory approaches lies in their inability to selectively control pathological inflammation while preserving antimicrobial defense capacity. Moreover, sepsis inflammation is highly redundant and heterogeneous, so neutralizing a single cytokine axis often fails to extinguish the broader pathological network while simultaneously perturbing protective immune signaling.

Therefore, there is an urgent need for a more refined therapeutic strategy, one that simultaneously suppresses pro‐inflammatory cascades, restores immune cell function, and maintains effective antimicrobial capacity through cell‐intrinsic mechanisms. Cell engineering strategies, particularly macrophage based immunotherapies, have emerged as promising approaches for treating immune dysregulatory diseases [15, 16, 17]. Macrophages, as key regulators of innate immunity and tissue homeostasis, possess remarkable plasticity and can be engineered to execute specific therapeutic functions [18]. Chimeric antigen receptor macrophages demonstrate this approach: by converting TNF‐α binding into IL‐4 signaling, CAR‐M autonomously polarizes to an M2 phenotype at inflamed sites and spontaneously deactivate upon inflammation resolution, reducing tissue damage in preclinical kidney and liver injury models without systemic immunosuppression [19].

mRNA delivery systems, particularly lipid nanoparticles (LNPs), provide unprecedented advantages for cellular engineering [20]. LNPs enable transient intracellular expression of therapeutic proteins with favorable tolerability and can be actively targeted to defined immune populations using antibody decoration, thereby reducing off‐target effects [21, 22, 23]. We selected an SM‐102‐based LNP because SM‐102 is a clinically validated ionizable lipid used in mRNA‐LNP vaccines for systemic nucleic‐acid delivery [24, 25]. Moreover, our LNP surface chemistry relies on PEG‐lipid components (DSPE‐PEG2000‐maleimide) for F4/80 antibody conjugation, which is compatible with standard SM‐102‐based LNP formulations used for mRNA transfection.

The sepsis microenvironment is characterized by immune dysregulation, wherein excessive pro‐inflammatory responses occur in parallel with subsequent immune suppression. Current CAR‐M strategies predominantly focus on bacterial clearance but fail to simultaneously address the dysregulated inflammation that constitutes the pathological hallmark of sepsis [26]. Preclinical evidence suggests that IL‐6 blockade can mitigate inflammatory injury in sepsis models, establishing IL‐6 as a promising therapeutic target [27]. However, single cytokine neutralization may be insufficient to fully restore immune homeostasis in the complex sepsis microenvironment [28, 29]. In contrast to conventional CAR‐M approaches that rely on highly specific receptor architectures to target individual pathogens, a strategy intervening upstream in the inflammatory cascade at an earlier stage, prior to full‐blown cytokine storm establishment, while simultaneously enabling broad‐spectrum antimicrobial defense through secreted effector antimicrobial peptides, may more faithfully recapitulate the complex immunopathological demands encountered in clinical sepsis. Such an approach circumvents the inherent constraints of single‐target CAR design and offers a therapeutically versatile framework that aligns with the heterogeneous and rapidly evolving nature of sepsis pathophysiology.

We hypothesized that simultaneous engagement of dual pathways blocking IL‐6 mediated pro‐inflammatory signaling while activating IL‐4 mediated anti‐inflammatory pathways would achieve superior bidirectional immune modulation compared to single‐target interventions. This rationale inspired our design of an IL‐6/4 chimeric fusion protein as a mechanistically optimized immunoregulatory molecule that simultaneously suppresses pathogenic inflammation and promotes tissue‐protective immunity. Concurrently, incorporation of the LL37 antimicrobial peptide would enhance the host's capacity to combat pathogens and confer broad‐spectrum antimicrobial capability. Thus, engineered macrophages co‐expressing the IL‐6/4 chimeric fusion protein and LL37 antimicrobial peptide represent a novel approach that simultaneously addresses two critical therapeutic deficiencies in current sepsis management: (1) immune dysregulation control through coordinated anti‐inflammatory and tissue‐protective signaling, and (2) broad‐spectrum antimicrobial capacity without dependence on single pathogen targeting.

Accordingly, in this study we utilized LNP‐mediated mRNA delivery to construct bifunctional engineered macrophages that simultaneously integrate the IL‐6/4 chimeric receptor architecture and enhanced LL37 antimicrobial peptide secretion. We systematically evaluated the biodistribution and cellular selectivity of LNP‐mediated in situ transfection, the immunomodulatory efficacy of the IL‐6/4 chimeric protein, the antimicrobial capacity of the LL37 peptide, and the synergistic therapeutic potential in preclinical sepsis models.

## 2 Materials and Methods

### 2.1 Mice

Wild‐type C57BL/6 mice (6–8 week old male and female) were purchased from Shanghai Yishang Biotechnology Co., Ltd. The mice were housed under pathogen‐free conditions at Shanghai Yishang Biotechnology Co., Ltd. All of the mice were maintained at a standard room temperature of approximately 25°C, with a 12 h light–dark cycle and humidity levels ranging from 40% to 60%, and were provided with normal chow. All animal procedures were approved by the Animal Care and Use Committee (IACUC) (protocol no. IACUC‐2025‐1‐024) and performed in accordance with relevant institutional guidelines. All animal experiments adhered to the animal life protection standards and procedures sanctioned by Shanghai Rat & Mouse Biotech Co., Ltd (Issue No. IACUC‐2025‐1‐024).

### 2.2 Cell Culture

RAW 264.7 cells were cultured in adherent cell culture plates in Iscove's Modification of Dulbecco's Modified Eagle Medium (IMDM, Corning) supplemented with 10% fetal bovine serum (FBS; Gibco), penicillin (100 IU/mL) and streptomycin (100 µg/mL). For retrieval of bone marrow‐derived macrophages (BMDMs), bone marrow was harvested from C57BL/6 mice by flushing the femur and tibia with 10 mL MACS buffer (Miltenyi Biotec). For red blood cell lysis, 1 mL of desalted water was added to the cell pellet, and immediately afterward, 50 mL MACS buffer was added. Cells were cultured in macrophage culture medium composed of RPMI medium (Corning) supplemented with 10% FBS, penicillin (100 IU/mL), streptomycin (100 µg/mL), and 50 ng/mL of mouse M‐CSF (Miltenyi Biotec). After seven days, 1 × 10^6^ BMDMs were seeded into a 24‐well plate and transduced with different mRNAs by using LNPs. We added to the cell culture medium either, for M2‐like polarization, 50 ng/mL of mouse IL‐4 (Miltenyi), three days after induction of polarization, flow cytometry (FC) analysis was performed.

### 2.3 Macrophages Culture with Soluble IL‐6

Macrophages were stimulated with soluble IL‐6 (50 ng/ml) at indicated concentrations for 24 h, with medium alone used as a control. The phenotype of CAR‐Ms was subsequently evaluated by flow cytometry, ELISA, and quantitative PCR (qPCR).

### 2.4 Infection and Treatment Model

Staphylococcus aureus ATCC 43300 was purchased from ATCC, the bacterial suspension was diluted 1:100 after shaking (200 rpm) overnight at 37°C, and shaken for another 4 h to allow the bacteria to be in the logarithmic growth phase. Bacteria were washed with PBS and resuspended in sterile PBS for inoculation. Mice were infected by intraperitoneal injection(i.p.) of *S. aureus* ATCC 43300 at a total dose of 1 × 10^8^ CFU. After 3 h of infection, Mice in the treatment group received intraperitoneal (i. v.) injections of IL‐6 antibody, LNP‐IL6/4Ra, or LNP‐IL6/4Ra‐LL37 (40 µg), while mice in the vehicle group received PBS via i.v. at the same time point. After 24 h of infection, mice were euthanized for cytokine assays, histological analysis, bacterial burden determination, liver and kidney function assessment, complete blood count analysis, and flow cytometric analysis of macrophage polarization. At day 3 post‐infection, mice were euthanized for flow cytometric analysis of T cell exhaustion.

### 2.5 The Cecal Ligation and Puncture (CLP) Model

A clinically relevant mouse model of sepsis was established using cecal ligation and puncture (CLP). Briefly, mice were anesthetized by intraperitoneal injection of 200 µL of 4% chloral hydrate (MACKLIN, China). After adequate anesthesia, a midline abdominal incision was made to expose the cecum. The cecum was gently exteriorized, ligated 1.0 cm from the distal end, and punctured once with a 22‐gauge needle. The cecum was then carefully repositioned into the abdominal cavity, and the abdominal incision was closed with sutures.

### 2.6 Blood Collection and Analysis

Venous blood samples were obtained from conscious mice via two alternative cannulation sites: the caudal vein or the orbital venous plexus. Fresh whole blood was immediately subjected to automated cell enumeration using the ProCyte DX hematology analyzer (IDEXX). For plasma collection, Blood specimens were transferred into anticoagulant‐containing BD Microtainer tubes (Becton Dickinson) and centrifuged at 3000 rpm for 10 min at ambient temperature. The acellular supernatant (plasma) was carefully separated from the sedimented blood cells and retained for subsequent analyses, while the cellular pellet was discarded. Serum was prepared by allowing whole blood to spontaneously clot for 40 min at room temperature in standard microcentrifuge tubes, followed by centrifugation at 3000 rpm for 10 min. The acellular fraction containing serum was collected after removing the coagulated material and cellular sediment (platelets, erythrocytes, and leukocytes). Serum alanine aminotransferase (ALT) and aspartate aminotransferase (AST) concentrations were quantified using commercially available enzyme assay kits on an automated chemistry platform (Shandong Bokon Biological Industry Co., Ltd.).

### 2.7 Cytokine Assay

For the cytokine assays, we utilized ELISA kits provided by Shanghai Jonlnbio Industrial Co., Ltd.(mouse TNF‐α; mouse IL‐6; mouse IFN‐γ; mouse IL‐12; mouse IL‐1β; mouse IL‐10; human IL‐10; human IL‐6; human IL‐1β) to measure cytokine levels in serum. As previously mentioned, after 12 h of infection, the peripheral blood was collected for analysis. The concentrations of IFN‐γ, TNF‐α, IL‐12, and IL‐6 in the serum were determined using standard ELISA procedures. An automated ELISA plate reader set to 450 nm was employed to measure and calculate the cytokine concentrations.

### 2.8 Bacterial Burden Determination

Bacterial loads at 24 h post‐infection were quantified from liver, spleen, lung, and kidney. Tissue samples were weighed and homogenized in 1–2 mL of sterile PBS. Culture supernatants were collected separately without homogenization. All samples were serially diluted using appropriate dilution factors to obtain countable colonies and plated on TSA plates. Plates were incubated at 37°C for 48 h, and bacterial colonies were quantified using ImageJ.

### 2.9 Histological Analysis

For histological analysis, the mice were euthanized after day 1 of infection as previously mentioned. Liver, kidney, spleen, and lung designated for histopathological assessment were collected following euthanasia and subjected to fixation in 10% neutral buffered formalin. Fixed tissues were subsequently dehydrated, cleared, and infiltrated with molten paraffin wax before embedding to generate solid paraffin blocks. Serial tissue sections measuring 3 micrometers in thickness were obtained using a microtome and transferred to positively charged glass slides. Sections were stained using the classical hematoxylin and eosin (H&E) technique in accordance with established OECD Good Laboratory Practice (GLP) guidelines and institutional standard operating procedures, ensuring adherence to principles of data integrity and analytical rigor. Histopathological evaluation was conducted by a board‐certified pathologist with extensive experience in morphological tissue analysis. Digital conversion of stained slides was performed using an automated histology slide scanner (Affinity Biosciences Panoramic 250 FLASH) at 20 × objective magnification. Subsequently, all digitized images underwent comprehensive review and diagnostic interpretation by an expert pathologist employing image analysis software.

### 2.10 Immunofluorescence Analysis

Tissue specimens were fixed in 4% paraformaldehyde (PFA; Sigma–Aldrich) in phosphate‐buffered saline (PBS) for 4–12 h at 4°C, depending on tissue size. After fixation, tissues were dehydrated through a graded ethanol series, cleared in xylene, and embedded in paraffin. Paraffin blocks were sectioned at 4–5 µm using a microtome, and the sections were mounted on glass slides.

Before staining, paraffin sections were deparaffinized in xylene and rehydrated through a graded ethanol series to water. Antigen retrieval was performed by heating the slides in a 95°C water bath for 20 min using either citrate buffer (10 mm citric acid, pH 6.0) or Tris‐EDTA buffer (10 mm Tris‐HCl, 1 mm EDTA, and 0.05% Tween‐20, pH 9.0). Slides were then allowed to cool in the retrieval buffer for 15 min at room temperature and rinsed three times with PBS.

To block nonspecific binding, sections were incubated in blocking buffer containing 5% normal donkey serum, 1% bovine serum albumin (BSA; Sigma‐Aldrich), and 0.3% Triton X‐100 in PBS for 1 h at room temperature. When mouse primary antibodies were used, an additional mouse IgG blocking step (Vector Laboratories) was performed according to the manufacturer's instructions.

Sections were then incubated overnight at 4°C with primary antibodies diluted in blocking buffer (CD68, Abclonal, 1:200; GFP, Abcam, 1:400). After primary antibody incubation, slides were washed five times with PBS containing 0.3% Triton X‐100. Appropriate secondary antibodies diluted in blocking buffer were applied for 1 h at room temperature in the dark, followed by six washes with PBS containing 0.3% Triton X‐100.

Nuclei were counterstained with DAPI (Beyotime; 1:1000 in PBS) for 5 min. After three additional washes with PBS, slides were mounted with Fluoromount‐G (Yeasen). Fluorescence images were acquired using a Leica SP8 Lightning confocal microscope (Leica Microsystems).

### 2.11 Quantitative Real‐Time PCR

The mRNA expression levels in BMDM cells, human monocytes were determined by real‐time PCR. The total RNA was extracted using a RNeasy micro kit (Qiagen) and subsequently reverse‐transcribed to cDNA using a QuantiTect reverse‐transcription kit, according to the manufacturer's instructions. Quantitative real‐time PCR was performed using 2 × TB Green Premix Ex Tavern II (Takara) on a QuantStudio 7 Flex Real‐Time PCR System (Thermo Fisher Scientific). The determination of cycle threshold values for each gene was followed by normalization to the internal reference gene β‐actin or GAPDH. The primers of interest are shown in Table S1.

### 2.12 Flow Cytometry

#### 2.12.1 Cell Suspensions Preparation

Processing of organs for FC analysis, organs were cut into small pieces and incubated with a tissue digestion solution composed of 1 mL IMDM (Corning) supplemented with 0.35 mg/mL collagenase type IV (from Clostridium histolyticum, Sigma–Aldrich), 1 mg/ml dispase II (Gibco), and 0.2 mg/ml DNAse (Roche) were added. Tissue digestion solution was then incubated at 37°C under agitation at 350 rpm for 10 min. The tissue was then further dissociated by pipetting and filtered using 0.04 mm cell strainers (Corning).

#### 2.12.2 Antibodies and Staining

For all cell populations, 0.5 × 10^6^ to 1 × 10^6^ cells were used for staining, with Fc block/anti‐CD16/32 (93, BioLegend) for blocking. Macrophages were sorted by CD11b and F4/80(M1/70; BM8, Biolegend), and T cells were sorted by CD3, CD4, CD8(17A2; GK1.5, Biolegend). Mouse CD206 and CD86(MR5D3, BD biosciences;PO3, Biolegend), human CD163 and CD86 were used for M1 and M2 macrophage polarization analysis (GHI/61, BU63, Biolegend), anti‐STAT6(pSTAT6 (CHI2S4N, Thermo Fisher) and pAKT(J1‐223.371, BD biosciences) were used for phosphorylation analyses. For pSTAT6 and pAKT staining, cells were fixed with methanol (−20°C) for 10 min and stained with intracellular markers. PD‐1 and Tim‐3 were used for T cell exhaustion markers (29F.1A12; B8.2C12, Biolegend).

#### 2.12.3 Cytotoxicity Assays

For the cytotoxicity assays, 1 × 10^6^ BMDMs were seeded in plates and incubated for 12 h. The culture medium was then replaced with fresh medium containing IL‐6Ra‐LNPs, IL‐6/4Ra‐LNPs, and IL‐6/4Ra‐LL37‐LNPs, followed by 48 h incubation. 1 × 10^6^ 43300 were added to the culture medium. For the CCK‐8 assay, detection kits were used according to the manufacturer's instructions, and data were collected.

#### 2.12.4 Bioluminescence Imaging

Male C57BL/6 mice, 6–8 weeks old, were used for in vivo luciferase expression studies. Luciferase mRNA‐lipid nanoparticle complexes were administered via intraperitoneal injection at a dose of 20 µg mRNA per mouse, diluted in PBS to a total volume of 200 µL. At designated time points, including 6, 12, 24, and 48 h post‐injection, mice were anesthetized with isoflurane, 3% for induction and 1%–2% for maintenance. D‐luciferin sodium salt (Yeasen) was then administered via intraperitoneal injection at a dose of 150 mg/kg. After 10–15 min to allow substrate distribution, whole‐body bioluminescence imaging was performed using an in vivo imaging system.

Based on the in vivo imaging results, the strongest bioluminescence signal was observed at 12 h post‐injection. Therefore, mice at this time point were rapidly euthanized after D‐luciferin administration and whole‐body imaging. Major organs, including the heart, liver, spleen, lungs, and kidneys, were harvested immediately for ex vivo bioluminescence imaging. The bioluminescence signals were quantified as total flux.

#### 2.12.5 mRNA Construction

The mRNA used in our work was customized by GenScript Corporation. The IL‐6/4Ra was engineered by combining the extracellular structural domains of the IL‐6Ra stalk and the IL‐4Ra intracellular domains, bridged by the IL‐6Ra transmembrane domain. The P2A and LL37 domains in IL6/4Ra‐LL37 were sequentially linked after the IL‐4Ra intracellular domains.

#### 2.12.6 LNP Formulation

Lipid nanoparticles (LNPs) were prepared using SM‐102 (38.9 mm; Aitowei (Shanghai) Pharmaceutical Technology Co., Ltd.; S042502), DSPC (10 mm; Aitowei (Shanghai) Pharmaceutical Technology Co., Ltd.; C20111), cholesterol HP (20 mm; Aitowei (Shanghai) Pharmaceutical Technology Co., Ltd.; C40311), DMG‐PEG2000 (1 mm; Aitowei (Shanghai) Pharmaceutical Technology Co., Ltd.; 241091), and DSPE‐PEG2000‐maleimide (0.5 mm; Nanosoft Biotechnology LLC; Lot 20490227). All lipids were dissolved in ethanol to generate a homogeneous lipid solution at a defined total lipid concentration.

#### 2.12.7 LNP Preparation

LNPs were formed by rapid mixing of the lipid‐containing ethanol phase with an aqueous phase consisting of 25 mm sodium acetate buffer (pH 4.0). Mixing was performed at a volumetric flow ratio of 1:3 (ethanol: aqueous) using a microfluidic mixer (or rapid injection under constant stirring).

Immediately after particle formation, the suspension was diluted with phosphate‐buffered saline (PBS, pH 7.4) and subjected to buffer exchange by centrifugal ultrafiltration (100 kDa molecular weight cutoff) to remove residual ethanol and adjust the pH.

#### 2.12.8 Antibody Thiolation

Purified monoclonal antibodies were thiolated using 2‐iminothiolane (Traut's reagent). Antibodies were incubated with Traut's reagent at a defined molar ratio in PBS (pH 8.0) for 1 h at room temperature with gentle mixing.

Excess reagent was removed by desalting, and the concentration of thiolated antibody was determined prior to conjugation.

#### 2.12.9 Antibody Conjugation to LNPs

For antibody conjugation, LNPs containing DSPE‐PEG2000‐maleimide were incubated with thiolated antibodies in PBS (pH 6.8–7.0). The reaction was carried out at room temperature for 2 h under gentle agitation to allow maleimide–thiol coupling. Unconjugated antibodies were removed by centrifugal ultrafiltration (100 kDa molecular weight cutoff). Antibody‐conjugated LNPs were resuspended in sterile PBS for subsequent experiments.

#### 2.12.10 Physicochemical Characterization

The hydrodynamic diameter and polydispersity index (PDI) of LNPs before and after antibody conjugation were measured by dynamic light scattering. Zeta potential was determined by electrophoretic light scattering.

Antibody conjugation efficiency was assessed by quantifying unbound antibodies in the filtrate using a bicinchoninic acid (BCA) protein assay.

### 2.13 Statistical Analysis

All experiments were performed with a minimum of three biological replicates. Data analysis was performed using GraphPad Prism version 10 (GraphPad Software Inc., USA). For comparisons among more than two groups, we used one‐way analysis of variance (ANOVA) followed by Tukey's multiple comparisons test or Dunnett's multiple comparisons test. Two‐group comparisons were conducted using unpaired 2‐tailed Student's t tests. All values are expressed as the mean ± SEM. P values less than 0.05 were considered to indicate statistical significance (^∗^ *p* < 0.05, <0.01, or ^∗∗∗^ *p* < 0.001). Detailed method information and the number of biological replicates are provided in the corresponding figure legends.

### 2.14 Others

Schematic created with BioRender.com (Agreement number: ML26FL6QFY).

## 3 Results

### 3.1 Therapeutic Efficacy of IL‐6 Antibody Treatment in Murine Sepsis Model

To validate the therapeutic efficacy of IL‐6 neutralization during acute‐phase sepsis, we established a Staphylococcus aureus (*S. aureus*) sepsis murine model (Figure 1A). C57BL/6 mice were subjected to intraperitoneal inoculation with 1 × 10^8^ CFU of *S. aureus* to induce sepsis. Anti‐IL‐6 antibody was administered intravenously 3 h post‐infection, with survival data recorded through day 7. Kaplan‐Meier survival analysis revealed that PBS‐treated control mice displayed approximately 90% mortality within 2 days, whereas anti‐IL‐6 antibody‐treated animals demonstrated markedly improved survival rates (Figure 1B), establishing the therapeutic efficacy of IL‐6 neutralization. Body weight monitoring provided additional evidence of disease severity and treatment efficacy (Extended Data Figure S1A).

**FIGURE 1:**
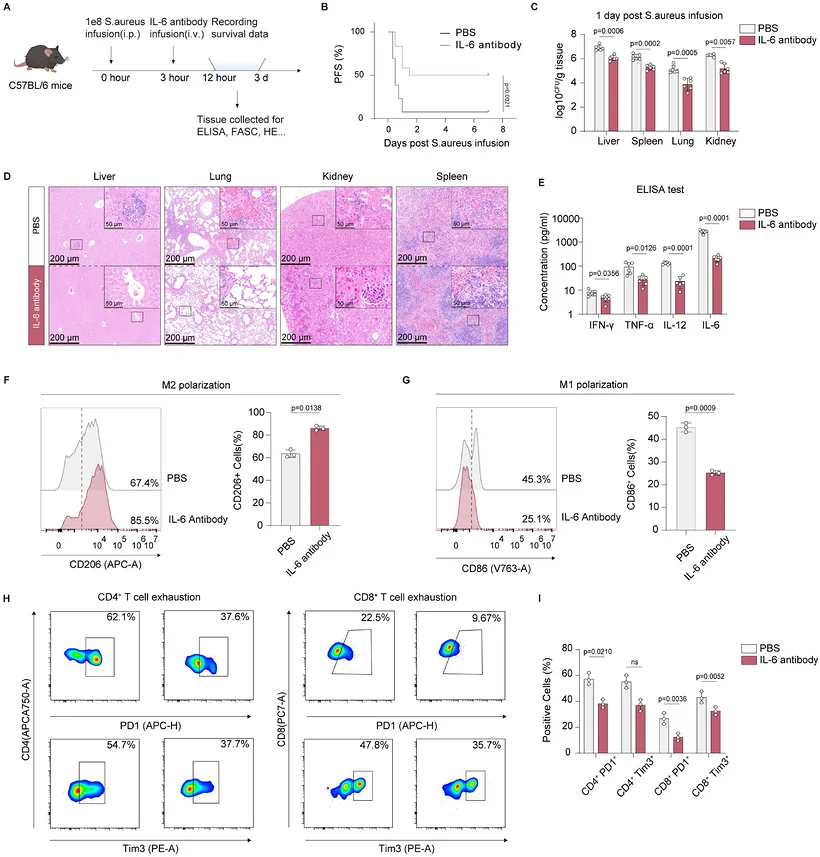
Therapeutic efficacy of IL‐6 neutralization in a murine sepsis model. (A) Schematic timeline of *S. aureus* sepsis induction and anti‐IL‐6 antibody treatment. (B) Kaplan‐Meier survival curves comparing PBS control and anti‐IL‐6 antibody‐treated mice through 7 days post‐infection (*n* = 12). (C) Quantification of bacterial colony‐forming units (CFU) in liver, spleen, kidney, and lung at 24 h post‐infection, demonstrating reduced bacterial burden in IL‐6 antibody‐treated mice (*n* = 6). (D) Representative hematoxylin and eosin (H&E)‐stained tissue sections from liver, lung, kidney, and spleen showing attenuated inflammatory cell infiltration and preserved tissue architecture in antibody‐treated animals. (E) Serum cytokine concentrations (IL‐6, TNF‐α, IL‐12, IFN‐γ) at 24 h post‐infection, demonstrating comprehensive suppression of pro‐inflammatory cytokine production (*n* = 6). (F,G) Flow cytometric analysis of M2 (CD206^+^) and M1 (CD86^+^) macrophage phenotypes at day 1 post‐infection, revealing enhanced M2 polarization frequency and reduced M1 activation. (H‐I) Quantification of PD‐1^+^ and Tim‐3^+^ expression on CD4^+^ and CD8^+^ T cells at day 3, demonstrating substantial rescue of T cell exhaustion markers (*n* = 3).

At 24 h post‐infection, bacterial burdens in major organs were quantified. IL‐6 antibody treated mice exhibited reduced bacterial colony forming units (CFU) in the liver, lung, spleen, and kidney relative to PBS controls (Figure 1C), demonstrating that IL‐6 neutralization promotes bacterial clearance. Histopathological examination revealed that PBS‐treated control animals developed extensive inflammatory cell infiltration, tissue necrosis, and structural disorganization across liver, lung, kidney, and spleen (Figure 1D). By contrast, IL‐6 antibody‐treated animals exhibited substantially preserved tissue structure with markedly attenuated inflammatory infiltration and effective mitigation of organ injury.

Serum cytokine test at 1 day further confirmed the immunoregulatory capacity of anti‐IL‐6 antibody treatment (Figure 1E). Relative to PBS controls, IL‐6 antibody treated mice displayed the most profound reduction in serum IL‐6 concentration, accompanied by significantly decreased levels of pro‐inflammatory cytokines including TNF‐α, IL‐12, and IFN‐γ. These findings demonstrate that IL‐6 antibody effectively interrupts pro‐inflammatory signaling axes and attenuates the cytokine storm characteristic of sepsis pathophysiology.

Flow cytometric analysis at 1 day revealed that PBS‐treated controls possessed 67.4% CD206^+^ (M2 phenotype) macrophages, whereas IL‐6 antibody treated animals exhibited an increased frequency of M2 macrophages at 85.5% (Figure 1F). This indicates that IL‐6 blockade promotes macrophage polarization toward the tissue‐protective M2 phenotype. Conversely, the frequency of pro‐inflammatory M1 phenotype macrophages (CD86^+^) was significantly reduced from 45.3% in PBS controls to 25.1% in IL‐6 antibody‐treated mice (Figure 1G), further confirming that IL‐6 neutralization suppresses pro‐inflammatory macrophage activation and restores immune balance disrupted by cytokine dysregulation.

In the sepsis model, CD4^+^ T cells exhibited a pronounced exhaustion phenotype even at 3 days, while IL‐6 antibody application rescued exhaustion. The frequency of PD‐1^+^CD4^+^ T cells was reduced from 62.1% in PBS controls to 37.6% in IL‐6 antibody‐treated animals (Figure 1H), Tim‐3^+^CD4^+^ T cell frequency decreased from 54.7% to 37.7%. These data demonstrate that IL‐6 antibody ameliorates CD4^+^ T cell exhaustion. CD8^+^ T cell exhaustion was also rescued. The frequency of PD‐1^+^CD8^+^ T cells was significantly reduced from 22.5% in PBS controls to 9.67% in IL‐6 antibody‐treated mice (Figure 1I); Tim‐3^+^CD8^+^ T cell frequency decreased from 47.8% to 35.7%. Collectively, these findings provide compelling evidence that IL‐6 antibody plays a critical role in rescuing early‐phase T cell exhaustion in sepsis.

In summary, IL‐6 antibody mediates multi‐tiered intervention in sepsis by blocking IL‐6 driven pro‐inflammatory signaling, thereby improving survival, reducing bacterial burden, attenuating inflammatory organ injury, promoting macrophage M2 polarization, and restoring T cell function. These findings provide critical scientific evidence and define therapeutic targeting for developing bifunctional engineered macrophages incorporating IL‐6 capture and macrophage M2 phenotype promotion.

### 3.2 Construction of F4/80‐Targeted Lipid Nanoparticle (LNP) Delivery System

To achieve macrophage‐selective transgene delivery for engineering this critical effector cell in sepsis, we designed and constructed an F4/80 antibody modified lipid nanoparticle (LNP)‐based targeted delivery system (Figure 2A). The LNP formulation consisted of DSPC, SM102, DMG‐PEG2000, DSPE PEG2000‐MAL, and Cho‐HP [30]. Chemical conjugation was accomplished by coupling the maleimide moiety of DSPE PEG2000‐MAL with the thiol group of the F4/80 antibody, generating F4/80‐decorated LNPs (LNP‐F4/80).

**FIGURE 2:**
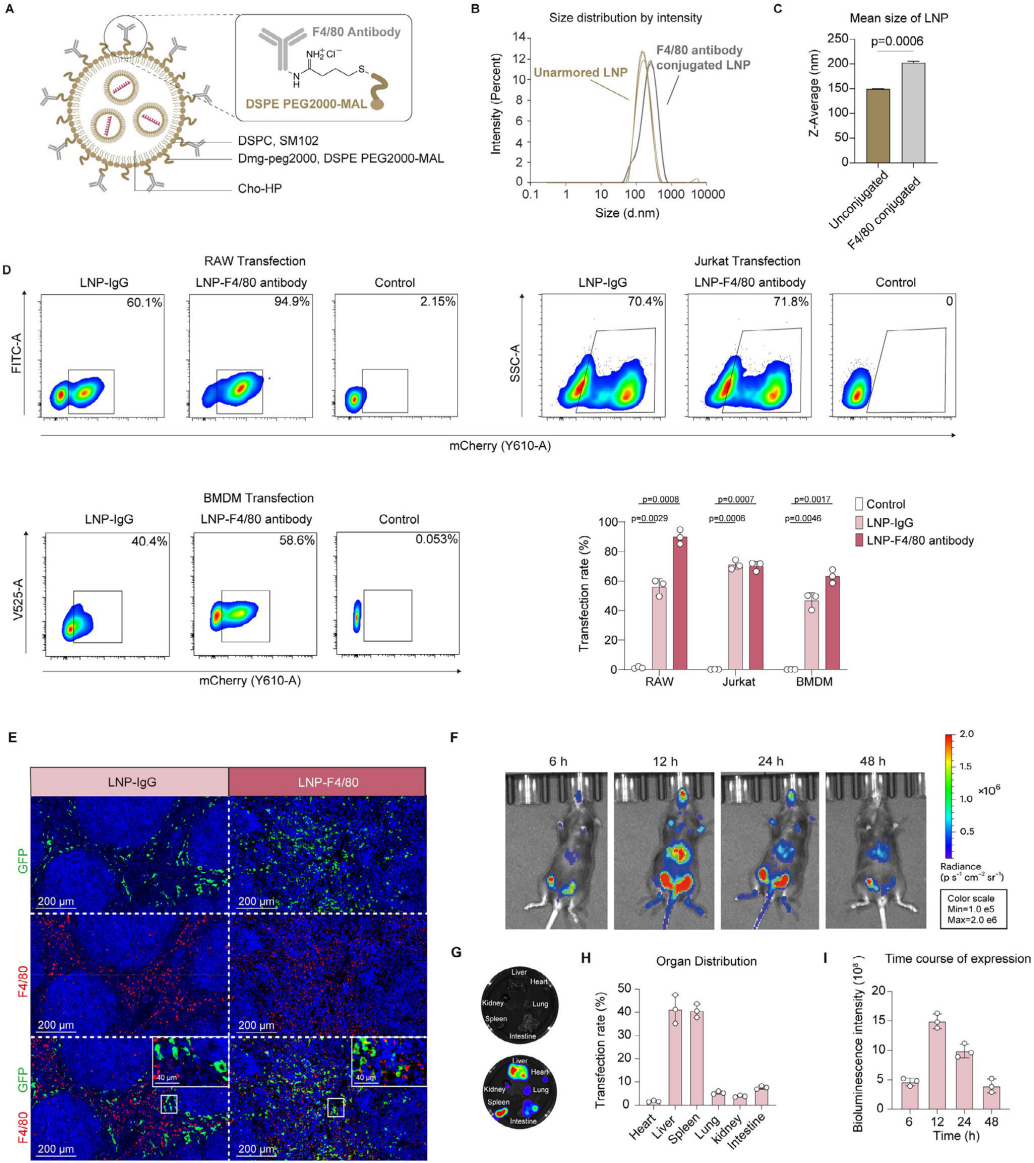
Design and characterization of F4/80‐targeted lipid nanoparticle delivery system. (A,B) Dynamic light scattering analysis showing size distribution patterns of unmodified and F4/80 antibody‐conjugated lipid nanoparticles (LNPs). (C) Quantitative analysis of mean particle diameter demonstrating significant size increase following F4/80 conjugation (unmodified: ∼150 nm; F4/80‐LNP: >200 nm). (D) Flow cytometric analysis of LNP‐F4/80 transfection efficiency in macrophages compared with isotype control LNPs (LNP‐IgG), and specificity validation in non‐macrophage cells (Jurkat T cells) (*n* = 3). (E) Immunofluorescence microscopy demonstrating extensive colocalization of GFP signal with F4/80^+^ macrophages in splenic tissue, compared to substantially weaker signals in LNP‐IgG control groups (*n* = 3). (F) In vivo bioluminescence imaging kinetics showing detectable luciferase signal at 6 h post‐injection with peak intensity at 24 h and sustained expression through 48 h post‐administration. (G,H) Organ biodistribution analysis revealing preferential LNP‐F4/80 accumulation in spleen and liver, with substantial fluorescent signal accumulation in lung and kidney—major sepsis‐affected organs. (I) Quantitative fluorescence intensity analysis demonstrating time‐dependent gene transfection dynamics with maximum expression at 24 h followed by sustained high‐level expression through the critical sepsis therapeutic window (*n* = 3).

Dynamic light scattering analysis demonstrated that unmodified LNPs and F4/80 antibody‐conjugated LNPs exhibited similar size distribution patterns (Figure 2B); however, F4/80 conjugation significantly increased mean particle diameter from approximately 150 nm for unmodified LNPs to over 200 nm (Figure 2C), reflecting successful antibody coupling.

To validate the macrophage identity and differentiation status, we performed phenotypic characterization of BMDMs (Extended Data Figure S2A). Flow cytometric analysis confirmed that BMDMs exhibited high‐level expression of F4/80, establishing successful macrophage differentiation and suitability for evaluating LNP‐F4/80 targeting specificity. Flow cytometric analysis evaluated the cell type selectivity of LNP‐F4/80 targeting capacity (Figure 2D). In macrophages, LNP‐F4/80 demonstrated substantially higher transfection efficiency compared to isotype control LNPs (LNP‐IgG), achieving transfection rates of 94.9% vs. 60.1% in RAW cells and 58.6% vs. 40.4% in BMDMs, respectively. By contrast, LNP‐F4/80 failed to enhance transfection efficiency in non‐macrophage cell lines (Jurkat T cells, 71.8% transfection vs. 70.4% for LNP‐IgG), conclusively demonstrating the macrophage‐specific targeting capability of this LNP system.

Immunofluorescence microscopy further confirmed the macrophage selectivity of LNP‐F4/80 (Figure 2E). In the spleen, green fluorescent protein (GFP) signal showed extensive colocalization with F4/80^+^ macrophages, indicating successful mRNA delivery into F4/80^+^ macrophage populations. In contrast, LNP‐IgG controls exhibited significantly reduced GFP/F4/80 co‐localization in the spleen, suggesting that non‐specific IgG modification fails to direct LNPs toward splenic macrophages, thereby underscoring the indispensable role of F4/80 antibody conjugation in macrophage‐targeted delivery.

In vivo bioluminescence imaging demonstrated that the LNP‐F4/80‐mediated fluorescent signal was readily detectable by 6 h post‐injection, with progressive signal intensity increases over time (Figure 2F). Peak signal was achieved at 24 h, with signal intensity beginning to decline by 48 h, indicative of favorable in vivo circulatory properties and sustained‐release kinetics. Organ biodistribution analysis revealed that LNP‐F4/80 preferentially accumulated in the spleen and liver (Figure 2G,H), representing the primary targeting organs. Notably, substantial fluorescent signal accumulation was also observed in the lung and kidney, effectively covering the major sepsis‐affected organs. Kinetic analysis of fluorescence intensity over time demonstrated pronounced time‐dependent gene transfection dynamics for LNP‐F4/80 (Figure 2I). An initial accumulation phase occurred 6–12 h post‐injection, with maximum expression achieved at 24 h, followed by sustained high‐level expression for 48 h thereafter, demonstrating that this LNP system maintains sufficient gene transduction efficiency throughout the critical therapeutic time window for sepsis treatment.

Transmission electron microscopy (TEM) was performed to assess the morphology of the prepared LNPs. LNP‐F4/80 particles exhibited predominantly spherical morphology with an electron‐dense core, supporting successful formulation of stable LNPs and antibody‐decorated LNPs for subsequent cellular engineering applications (Extended Data Figure S3A). Zeta potential analysis was performed to evaluate the surface charge and colloidal stability of the LNPs. The LNP formulation displayed a strongly negative zeta potential (mean ∼ −14.22 mV, SD 0.74 mV), consistent with the presence of negatively charged/sterically stabilized surface components and suggesting favorable colloidal stability for downstream biological applications (Extended Data Figure S3B). To evaluate the biosafety profile of the LNP delivery system, we performed comprehensive in vitro and in vivo toxicity assessments (Extended Data Figures S4–S6). In vitro cytotoxicity assays demonstrated that LNP‐GFP treatment did not compromise cell viability in BMDMs across multiple time points (0, 6, 12, and 24 h), with viability consistently exceeding 95% and showing no significant difference compared to untreated controls (Extended Data Figure S4). Biochemical analysis of hepatic and renal function markers revealed no evidence of organ toxicity following LNP administration (Extended Data Figure S5A,B). Serum levels of alanine aminotransferase and aspartate aminotransferase remained comparable between LNP‐treated and untreated groups, indicating preserved hepatic function. Similarly, renal function parameters including blood urea nitrogen and creatinine showed no significant alterations. Histopathological examination of major organs (liver, spleen, lung, and kidney) by hematoxylin and eosin (H&E) staining revealed normal tissue architecture with no evidence of inflammatory infiltration, necrosis, or structural damage in LNP‐treated mice compared to untreated controls (Extended Data Figure S6A). These findings collectively establish the excellent biocompatibility and safety profile of our LNP delivery system, supporting its suitability for in vivo therapeutic applications.

Collectively, the F4/80‐targeted LNP delivery system we successfully constructed demonstrates the following advantages: (1) pronounced macrophage‐specific recognition with transfection efficiency significantly exceeding irrelevant controls; (2) effective gene delivery achieved in immune hub organs (spleen, liver) as well as major sepsis‐affected organs (lung, kidney); (3) rational in vivo biodistribution kinetics with sustained high level gene expression maintenance throughout the critical sepsis therapeutic window. These characteristics conclusively establish the feasibility and efficacy of this LNP system for achieving in vivo macrophage engineering in murine sepsis models.

### 3.3 Construction of Chimeric IL‐6/4R Promoting Macrophage Repair Phenotype and IL‐6 Sequestration

Building upon our earlier findings demonstrating therapeutic efficacy of IL‐6 antibody treatment in sepsis and the high efficiency targeting capability of the LNP delivery system, and considering that chimeric cytokine receptors have been successfully applied in various diseases including cancer and autoimmune disorders [31, 32], we further designed and constructed a novel chimeric receptor (IL6/4Ra) by fusing the extracellular domain of the IL‐6 receptor (IL6Ra ECD) with the intracellular signaling domain of the IL‐4 receptor (IL4Ra ICD) (Figure 3A). This rational design aimed to simultaneously achieve two critical functions: selective IL‐6 capture and sequestration via the IL6Ra extracellular domain, while activating downstream signaling pathways promoting macrophage repair phenotype through the IL4Ra intracellular domain.

**FIGURE 3:**
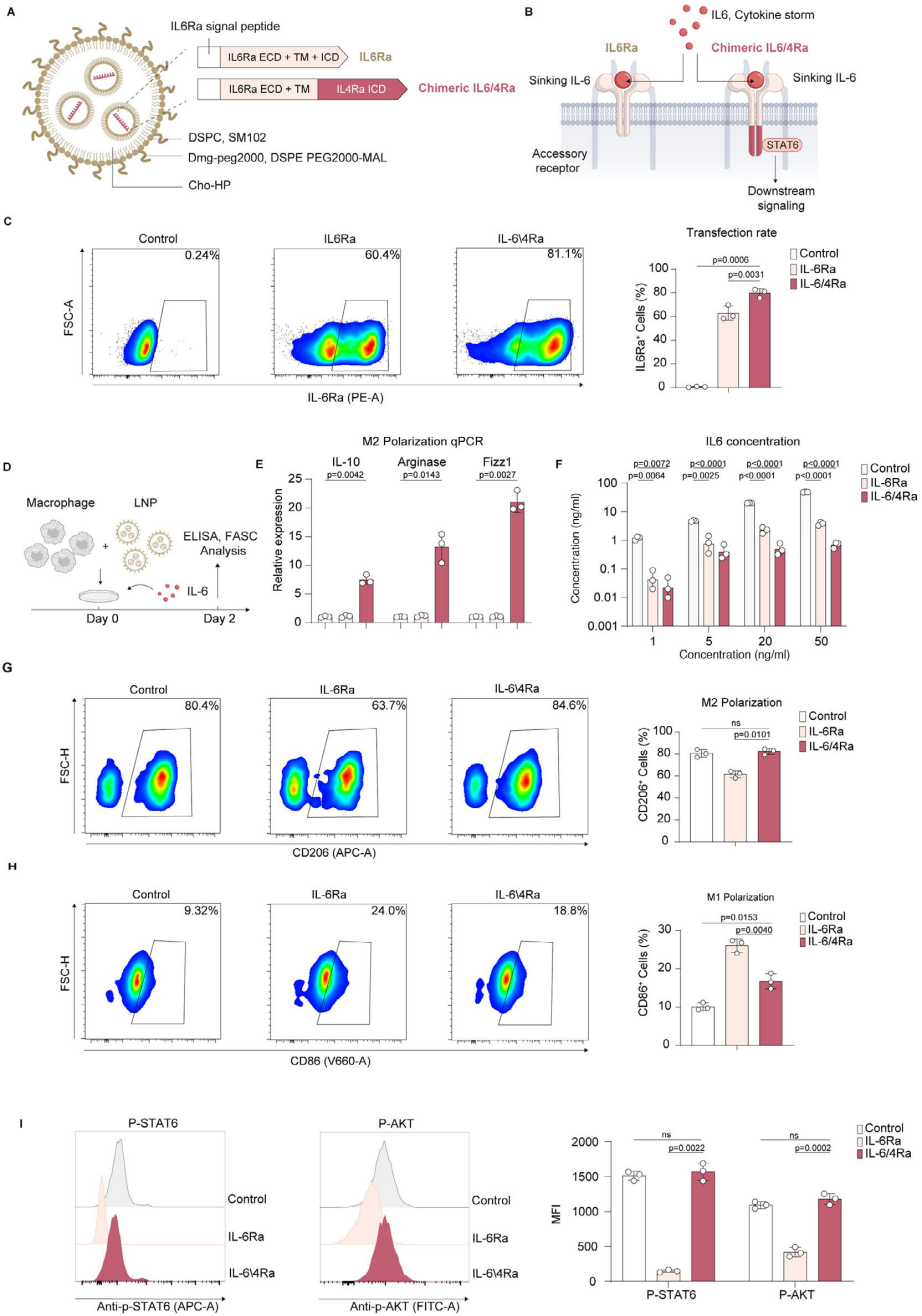
Chimeric IL‐6/4Ra promotes macrophage reparative phenotype and IL‐6 sequestration. (A,B) Schematic illustration of chimeric IL‐6/4Ra architecture combining the extracellular domain of IL‐6 receptor (IL‐6Ra ECD) with the intracellular signaling domain of IL‐4 receptor (IL‐4Ra ICD) linked by the IL‐6Ra transmembrane domain. (C) Flow cytometric analysis confirming successful transgene delivery into bone marrow‐derived macrophages (BMDMs). (D) Experimental design showing macrophage stimulation with 50 ng/mL soluble IL‐6. (E) Quantitative real‐time PCR analysis demonstrated markedly elevated expression of M2 phenotype‐associated genes (IL‐10, Arginase‐1, Fizz1) in IL‐6/4Ra‐expressing cells relative to controls and IL‐6Ra‐only cells. (F) ELISA quantification of IL‐6 concentration in culture supernatants under concentration‐dependent IL‐6 stimulation (1–50 ng/mL), demonstrating superior IL‐6 capture and sequestration capacity of the chimeric receptor. (G,H) Flow cytometric analysis of M2 (CD206^+^) and M1 (CD86^+^) macrophage marker frequencies, revealing enhanced M2 polarization and suppressed M1 phenotype in IL‐6/4Ra‐expressing cells. (I) Phosphoflow cytometry revealing significantly elevated phosphorylated STAT6 (P‐STAT6) and phosphorylated AKT (P‐AKT) levels in IL‐6/4Ra‐expressing cells, indicating activation of canonical M2 polarization signaling cascades.

Flow cytometric analysis confirmed successful delivery of IL‐6Ra and IL6/4Ra transgenes into macrophages using the LNP‐F4/80 delivery system, with transfection efficiencies of 60.4% and 81.1%, respectively (Figure 3C), demonstrating superior expression efficiency of the chimeric receptor. To evaluate the impact of the chimeric receptor on macrophage function, we stimulated in vitro‐transfected macrophages with 50 ng/mL IL‐6 (Figure 3D) and performed comprehensive analysis at day 2. Quantitative PCR revealed that macrophages expressing IL6/4Ra demonstrated markedly elevated expression levels of M2 phenotype‐associated genes relative to controls and IL‐6Ra‐only‐expressing cells: IL‐10, Arginase, and Fizz1 expression were all substantially upregulated (Figure 3E), confirming potent M2 polarization‐promoting activity of the chimeric receptor. Flow cytometric analysis further substantiated these findings. Using IL‐4‐treated macrophages as the control, the frequency of CD206^+^ M2‐like cells was 80.4% in the control group, decreased to 63.7% in IL‐6Ra‐expressing cells, and increased to 84.6% in IL6/4Ra‐expressing cells (Figure 3G), indicating that the chimeric receptor restored and maintained the M2 phenotype more effectively than IL‐6Ra alone. Consistently, the frequency of CD86^+^ M1‐like cells was 9.32% in the control group, increased to 24.0% in IL‐6Ra‐expressing cells, and decreased to 18.8% in IL6/4Ra‐expressing cells (Figure 3H), indicating that the chimeric receptor not only promoted M2 polarization but also partially suppressed M1 pro‐inflammatory activation relative to IL‐6Ra alone.

ELISA analysis revealed superior IL‐6 sequestration capacity of the chimeric IL6/4Ra receptor (Figure 3F). Under IL‐6 stimulation conditions, control culture supernatants displayed concentration‐dependent increases in IL‐6 levels across the gradient (1–50 ng/ml), and IL‐6Ra‐expressing cells demonstrated significantly reduced IL‐6 levels. Most critically, IL6/4Ra‐expressing cells maintained consistently low IL‐6 concentrations across all dose gradients tested, conclusively demonstrating the high‐efficiency IL‐6 sequestration and capture function of the chimeric receptor. Beyond IL‐6 neutralization, IL6/4Ra expression exerted broader immunomodulatory effects on macrophage polarization (Extended Data Figure S8A). ELISA quantification of pro‐inflammatory cytokines revealed that IL6/4Ra‐expressing BMDMs secreted significantly reduced levels of IL‐1β and tumor necrosis factor‐α. These findings indicate that the IL6/4Ra fusion protein not only sequesters IL‐6 but also suppresses the broader M1 type pro‐inflammatory program in macrophages. To elucidate the molecular mechanism of the chimeric receptor, we quantified levels of phosphorylated key signaling proteins (Figure 3I). Flow cytometric analysis revealed that IL6/4Ra‐expressing cells displayed substantially elevated mean fluorescence intensity (MFI) of phosphorylated STAT6 relative to IL‐6Ra‐expressing cells. This indicates that the chimeric receptor activates the canonical STAT6 signaling pathway through IL‐6/4Ra extracellular interaction. Additionally, phosphorylated AKT (P‐AKT) levels demonstrated upward trending as well in IL‐6/4R cells. This reflected activation of the PI3K/AKT signaling pathway, which is equally critical for M2 macrophage survival and functional maintenance.

To assess the clinical translational potential of the chimeric IL6/4Ra receptor, we validated its function in human‐derived macrophages. LNP‐mediated delivery of IL‐6Ra and IL6/4Ra transgenes resulted in flow cytometry‐determined transfection efficiencies of 2.05% (control), 23.6% (IL‐6Ra), and 41.9% (IL6/4Ra) (Extended Data Figure S7A). IL6/4Ra expression promoted an M2‐like polarization phenotype, increasing CD163+ M2‐like macrophages to 34.1% vs. 12.4% in the IL‐6Ra group (Extended Data Figure S7B) and reducing the CD86+ pro‐inflammatory population (23.9% vs. 28.8%; Extended Data Figure S7C).

Mechanistically, IL6/4Ra enhanced downstream signaling, with increased p‐STAT6 MFI and elevated p‐AKT levels compared with IL‐6Ra‐expressing cells (Extended Data Figure S7D). Consistent with this, ELISA showed that IL6/4Ra reshaped cytokine secretion toward an anti‐inflammatory profile, including increased IL‐10 (Extended Data Figure S7E). Under IL‐6 stimulation (1–50 ng/mL), IL6/4Ra‐expressing cells efficiently captured/sequestered IL‐6 and maintained low levels of IL‐6 in culture supernatants (Extended Data Figure S7F). Consistently, qPCR further confirmed upregulation of M2‐associated markers such as CCL18 and CD209 (Extended Data Figure S8B).

Collectively, these data conclusively establish the IL‐6 sequestration capacity and system compatibility of the chimeric receptor in human cellular systems.

### 3.4 Optimization of Antimicrobial Capability of LL37 Antimicrobial Peptide‐Fused Engineered Macrophages

Although IL‐6 neutralization and M2 polarization effectively control inflammation, promotion of the repair phenotype alone may compromise the intrinsic antimicrobial and bactericidal capacity of macrophages. LL‐37 (Leu‐Leu‐37) is the sole human cathelicidin antimicrobial peptide that exerts broad‐spectrum antimicrobial activity through direct membrane disruption [33]. As a cationic amphipathic peptide, LL‐37 electrostatically binds to negatively charged bacterial membranes, inserts into the lipid bilayer, and induces membrane permeabilization and lysis, resulting in rapid bacterial killing [34]. To maintain M2 repair function while simultaneously enhancing the antimicrobial efficacy of engineered macrophages, we incorporated the antimicrobial peptide LL37 into the chimeric IL6/4Ra structure (Figure 4A,B). ELISA analysis confirmed highly efficient expression and secretion of LL37 in engineered macrophages (Figure 4D). LL37 secretion concentration in IL6/4Ra‐LL37‐expressing cells was markedly elevated to approximately 10 ng/ml, representing several‐fold increases relative to controls and IL6/4Ra‐only expressing cells, indicating that incorporation of the LL37 sequence significantly enhances LL37 expression in macrophages.

**FIGURE 4:**
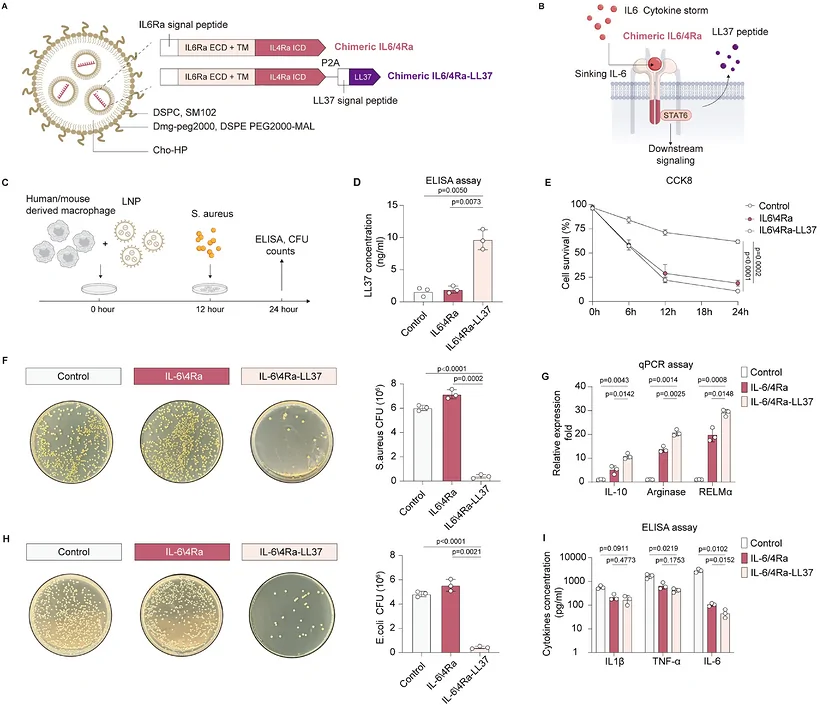
Optimization of LL37 antimicrobial peptide‐engineered macrophages for antimicrobial activity. (A,B) Schematic illustration of the IL‐6/4Ra‐LL37 fusion construct incorporating the LL37 antimicrobial peptide downstream of the IL‐4Ra intracellular domain, separated by a P2A self‐cleaving peptide linker to ensure independent translation. (C) Schematic overview of the experimental workflow. (D) ELISA quantification of secreted LL37 demonstrating approximately 10 ng/mL production in IL‐6/4Ra‐LL37‐expressing BMDMs with negligible levels in controls and IL‐6/4Ra cells (*n* = 3). (E) Cell viability assay (CCK‐8) during 24 h macrophage and *S. aureus* co‐culture demonstrated enhanced macrophage survival in IL‐6/4Ra‐LL37‐expressing cells compared to controls (*n* = 3). (F) Representative *S.aureus* bacterial colony plates from different treatment groups showing bacterial reduction with LL37 fusion. (G) Quantitative real‐time PCR analysis of M2 phenotype genes (IL‐10, Arginase‐1, RELMα) confirming maintained M2 polarization‐promoting function with comparable expression levels to IL‐6/4Ra‐expressing cells (*n* = 3). (H) Representative E. coli bacterial colony plates from different treatment groups showing bacterial reduction with LL37 fusion. (I) ELISA quantification of pro‐inflammatory (IL‐1β) and anti‐inflammatory (IL‐10, TNF‐α) cytokines demonstrating substantially reduced IL‐1β with appropriately regulated anti‐inflammatory factors (*n* = 3).

In vitro co‐culture experiments assessed macrophage viability following interaction with bacteria (Figure 4C,E). CCK‐8 analysis revealed that IL6/4Ra‐LL37‐expressing cells maintained substantially higher cell survival rates relative to controls throughout 24‐h co‐culture, indicating that LL37 incorporation not only avoided enhanced macrophage toxicity but rather reduced bacterial toxicity‐induced cellular damage through enhanced antimicrobial capability.

Direct colony enumeration of *S. aureus* and E. coli demonstrated substantially enhanced antimicrobial capacity of LL37‐fused engineered macrophages (Figure 4F,H). Control and IL6/4Ra‐only expressing cells maintained elevated bacterial colony counts, whereas IL6/4Ra‐LL37‐expressing cells demonstrated dramatically reduced colony counts, exhibiting extremely potent antimicrobial efficacy. These findings conclusively establish that LL37 incorporation effectively compensates for potential antimicrobial deficiency associated with M2 polarization. Notably, LL37 fusion did not compromise maintenance of polarization function in engineered macrophages (Figure 4G,I). Quantitative PCR demonstrated that M2 marker genes, including IL‐10, Arginase, and RELMα, maintained consistently elevated expression levels comparable to IL6/4Ra‐expressing cells in IL6/4Ra‐LL37‐expressing cells. Simultaneously, ELISA analysis revealed substantially reduced pro‐inflammatory factor IL‐1β, with anti‐inflammatory factors IL‐10 and TNF‐α levels demonstrating appropriate regulation, confirming intact polarization‐promoting function of the chimeric receptor.

To validate the translational potential of this approach, we further assessed the antimicrobial efficacy and cellular compatibility in human macrophages (Extended Data Figure S9). Consistent with murine macrophage results, IL6/4Ra‐LL37‐expressing human macrophages exhibited substantially elevated LL37 secretion (Extended Data Figure S9C), reaching approximately 14 ng/ml compared to negligible levels in control and IL6/4Ra groups. Direct bacterial enumeration demonstrated that IL6/4Ra‐LL37 treatment significantly reduced both *S. aureus* (Extended Data Figure S9A) and E. coli (Extended Data Figure S9B) colony counts, representing dramatic reductions compared to controls. Importantly, enhanced antimicrobial activity was accompanied by improved macrophage survival during bacterial co‐culture (Extended Data Figure S9D). IL6/4Ra‐LL37‐expressing cells maintained approximately 60% viability at 24 h, significantly higher than control (∼12%) and IL6/4Ra (∼15%) groups, demonstrating that LL37 fusion effectively protected human macrophages from bacterial toxicity induced damage. These results confirm that the therapeutic strategy is equally effective in human macrophage systems, supporting its clinical translational potential.

### 3.5 In Vivo Therapeutic Efficacy Assessment of Engineered Macrophages in Sepsis

To validate the therapeutic efficacy of LL37‐fused engineered macrophages in sepsis, we established a murine sepsis model with LNP‐F4/80‐mediated in situ gene transfection (Figure 5A). IL6/4Ra‐LL37 treatment markedly improved survival rates in septic mice (Figure 5B). In striking contrast to the rapid mortality observed in controls, IL6/4Ra‐LL37‐treated animals demonstrated a pronounced survival advantage. Body weight monitoring revealed that IL6/4Ra‐LL37‐treated mice exhibited accelerated body weight recovery, indicating substantially more pronounced overall clinical improvement (Figure 5C).

**FIGURE 5:**
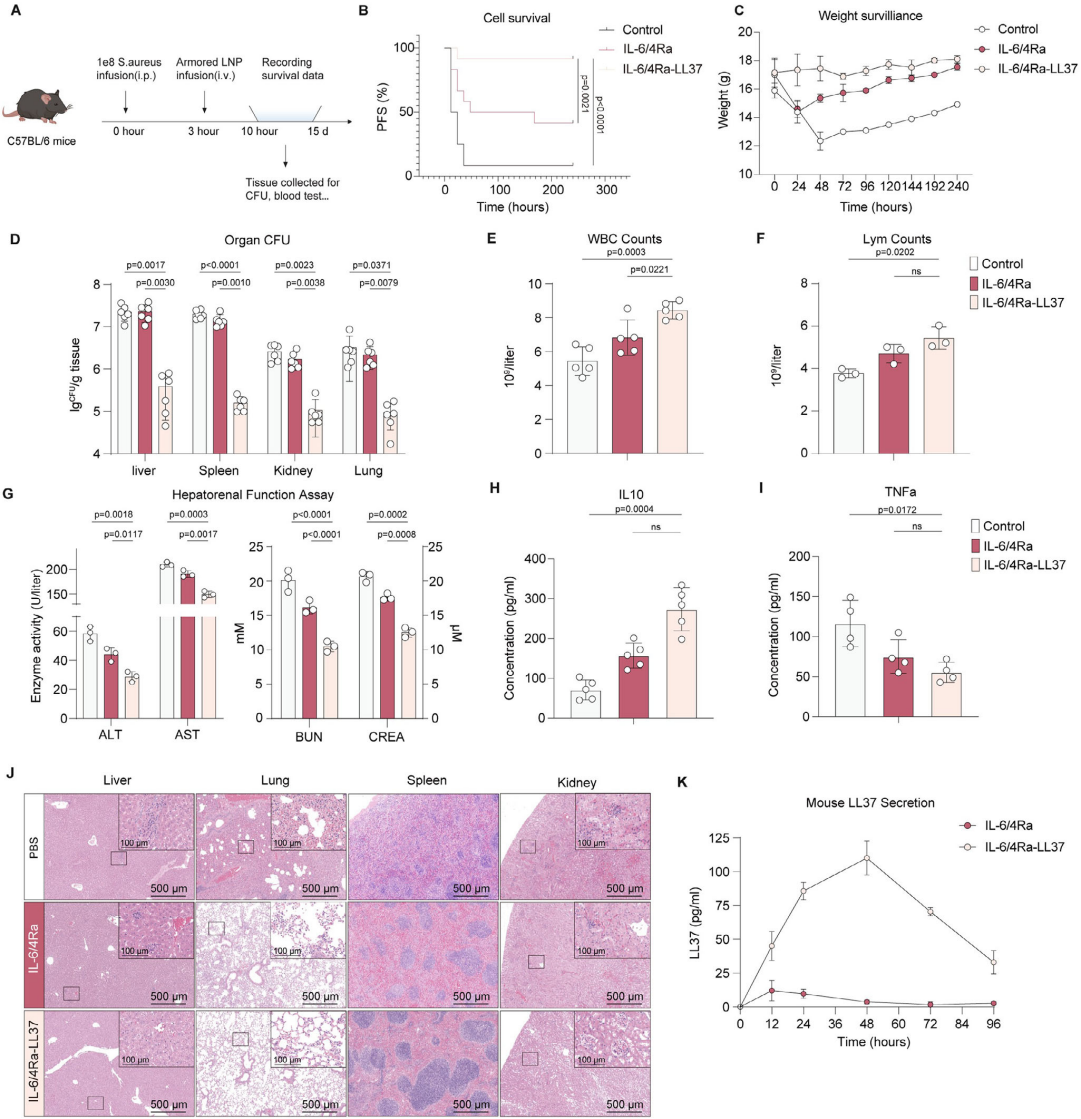
In vivo therapeutic efficacy of LL37‐fused engineered macrophages in sepsis. (A) Experimental timeline: C57BL/6 mice received intraperitoneal (i.p.) injection of 1 × 10^8^ CFU *S. aureus* ATCC 43300 at time 0 h, followed by intravenous (i.v.) injection of LNP‐F4/80‐IL‐6/4Ra‐LL37 at 3 h post‐infection (*n* = 12). (B) Kaplan‐Meier survival curves demonstrating markedly improved 7‐day survival rates in IL‐6/4Ra‐LL37‐treated mice (*n* = 12). (C) Body weight monitoring showing accelerated weight recovery in treated animals, indicating substantially enhanced overall clinical improvement (*n* = 8). (D) Quantitative organ bacterial burden analysis at 24 h post‐infection revealing significantly reduced CFU in liver, spleen, kidney, and lung following IL‐6/4Ra‐LL37 treatment (*n* = 6). (E,F) Complete blood count (CBC) analysis at 24 h post‐infection showing elevated white blood cell (WBC) and lymphocyte counts in treated animals, indicating successful correction of sepsis‐induced immune cell population abnormalities (*n* = 5). (G) Hepatorenal function biomarkers (alanine aminotransferase [ALT], aspartate aminotransferase [AST], blood urea nitrogen [BUN], creatinine [CREA]) assessed at 24 h demonstrating substantial amelioration of sepsis‐induced organ dysfunction, with most pronounced hepatic function recovery (*n* = 3). (H,I) Serum cytokine analysis at 24 h post‐infection showing substantially elevated anti‐inflammatory IL‐10 concentrations with appropriately controlled pro‐inflammatory TNF‐α and IL‐6 levels, indicating successful transition from pro‐ to anti‐inflammatory immune responses. (J) Representative H&E‐stained tissue sections from liver, lung, spleen, and kidney demonstrating substantially attenuated inflammatory cell infiltration and reduced tissue injury in IL‐6/4Ra‐LL37‐treated animals compared to control PBS‐treated mice. (K) Kinetic analysis of serum LL37 concentration reaching peak concentration of approximately 110  pg/mL at 48 h post‐treatment followed by gradual decline while maintaining therapeutically sufficient antimicrobial concentration (*n* = 3).

Quantitative organ bacterial burden analysis at 24 h post‐infection demonstrated that IL6/4Ra‐LL37 treatment significantly reduced bacterial CFU loads across multiple organs, including liver, spleen, kidney, and lung (Figure 5D). comprehensively demonstrated the enhanced antimicrobial capacity of LL37‐fused engineered macrophages. Complete blood count analysis at 24 h post‐infection revealed elevated white blood cell (WBC) counts in IL6/4Ra‐LL37‐treated animals (Figure 5E), with lymphocyte counts likewise substantially increased (Figure 5F), indicating that this therapeutic approach successfully corrected sepsis‐induced immune cell population abnormalities. Hepatic and renal function biomarkers assessed at 24 h demonstrated that sepsis induced significant organ injury with elevations in alanine aminotransferase (ALT), aspartate aminotransferase (AST), blood urea nitrogen (BUN), and creatinine (CREA) (Figure 5G). IL6/4Ra‐LL37 treatment substantially ameliorated these parameters, with most pronounced hepatic function recovery and significant improvements in renal function, demonstrating effective organ‐protective capacity of engineered macrophages against sepsis‐induced organ dysfunction.

Cytokine analysis assessed at 24 h further confirmed therapeutic efficacy (Figure 5H,I). IL6/4Ra‐LL37treated animals exhibited substantially elevated IL‐10 concentration, with TNF‐α levels appropriately controlled, indicating successful transition from pro‐inflammatory to anti‐inflammatory immune responses. Histopathological examination revealed that IL6/4Ra‐LL37 treatment substantially attenuated sepsis‐induced inflammatory infiltration and tissue injury across hepatic, pulmonary, splenic, and renal tissues (Figure 5J). Kinetic analysis demonstrated effective in vivo expression of IL6/4Ra‐LL37 delivered via LNP‐F4/80. LL37 reached peak serum concentration of approximately 110 pg/ml at 48 h post‐treatment, followed by gradual decline while maintaining therapeutically sufficient antimicrobial concentration (Figure 5K), completely encompassing the critical therapeutic time window for sepsis intervention.

In vivo flow cytometric analysis at 24 h further elucidated the capacity of IL6/4Ra‐LL37 to induce macrophage phenotype switching in situ(gating strategies shown in Extended Data Figure S10). M2 marker CD206^+^ cell frequency increased from 10.9% in controls to 28.0% in IL6/4Ra‐treated animals, with further elevation to 37.2% in IL6/4Ra‐LL37‐treated mice (Figure 6A,B). Correspondingly, M1 marker CD86^+^ cell frequency was substantially reduced from 20.5% in controls to 6.70% in IL6/4Ra‐LL37‐treated animals (Figure 6C,D), demonstrating that LL37 fusion not only helped to maintain but further strengthened M2 polarization efficacy. Blood immune cell analysis revealed that IL6/4Ra‐LL37 treatment substantially increased CD4^+^ and CD8^+^ T cell numbers (Figure 6E,F), while concurrently reducing neutrophil counts (Figure 6G) and appropriately regulating monocyte frequency (Figure 6H), thereby achieving more balanced immune cell composition (gating strategies shown in Extended Data Figure S11).

**FIGURE 6:**
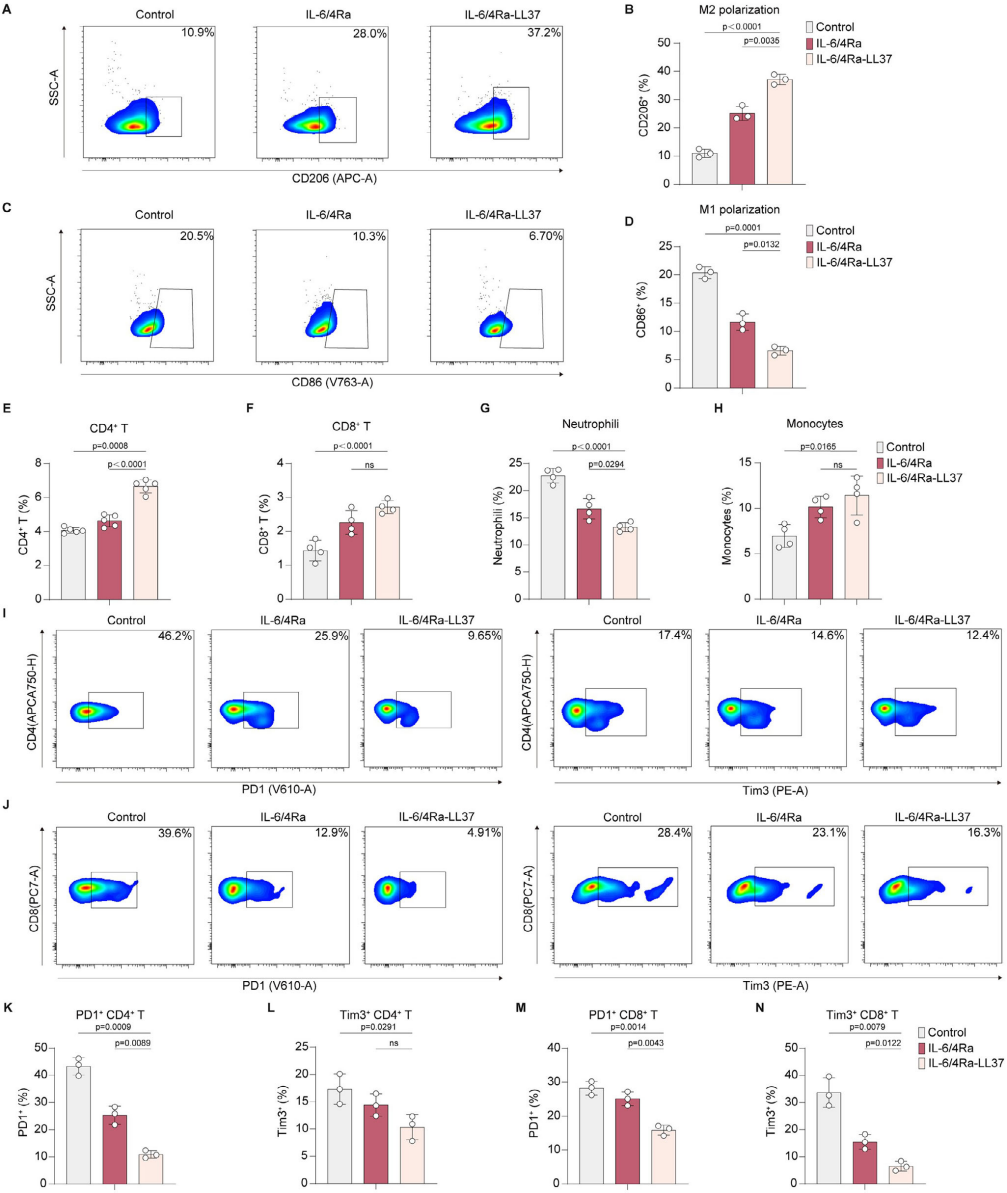
Immune cell phenotypic shift and functional recovery induced by engineered macrophages in sepsis. (A,B) Flow cytometric analysis of macrophage M2 (CD206^+^) phenotype frequency at day 1 post‐infection (*n* = 3). (C,D) Flow cytometric analysis of macrophage M1 (CD86^+^) phenotype frequency at day 1 post‐infection (*n* = 3). (E–H) The proportions of CD4^+^ T cells, CD8^+^ T cells, neutrophils, and monocytes in peripheral blood (*n* = 3). (I,J) Flow cytometric quantification of T cell exhaustion. (K,L) CD4^+^ T cell exhaustion marker analysis showing. (M,N) CD8^+^ T cell exhaustion marker analysis showing. All flow cytometry gating strategies are detailed in Extended Data Figures S10 and S11.

Analysis of T cell exhaustion markers at day 3 after infection showed marked upregulation of PD‐1 and Tim‐3 on both CD4^+^ and CD8^+^ T cells in control mice, indicating pronounced sepsis‐induced T cell exhaustion. In control animals, 46.2% of CD4^+^ T cells and 39.6% of CD8^+^ T cells were PD‐1^+^, while 17.4% of CD4^+^ T cells and 28.4% of CD8^+^ T cells were Tim‐3^+^. Treatment with IL6/4Ra‐LL37 substantially reduced these exhaustion‐associated populations, with PD‐1^+^ CD4^+^ and CD8^+^ T cells decreasing to 9.65% and 4.91%, respectively, and Tim‐3^+^ CD4^+^ and CD8^+^ T cells decreasing to 12.4% and 16.3%, respectively (Figure 6I–N). These findings indicate that engineered macrophages alleviated sepsis‐induced T cell exhaustion (gating strategies are shown in Extended Data Figure S12).

Collectively, these in vivo findings conclusively establish that LL37‐fused engineered macrophages simultaneously achieved inflammation control, M2 polarization promotion, T cell functional recovery, and enhanced antimicrobial capacity, providing critical in vivo evidence supporting clinical translation of this therapeutic approach.

### 3.6 In vivo Therapeutic Evaluation of IL‐6/4Ra–LL37 Engineered Macrophages in CLP Sepsis Model

To further evaluate the therapeutic potential of the engineered macrophages in a clinically relevant pathological setting, we established a cecal ligation and puncture (CLP) induced sepsis mouse model. F4/80‐targeted LNPs were used for in situ gene delivery. After CLP induction, mice were treated 3 h post‐surgery with targeted LNPs loaded with mRNAs encoding IL‐6/4Ra or IL‐6/4Ra–LL37, and survival, body weight changes, and multiple immunological and organ function parameters were monitored longitudinally (Figure 7A).

**FIGURE 7:**
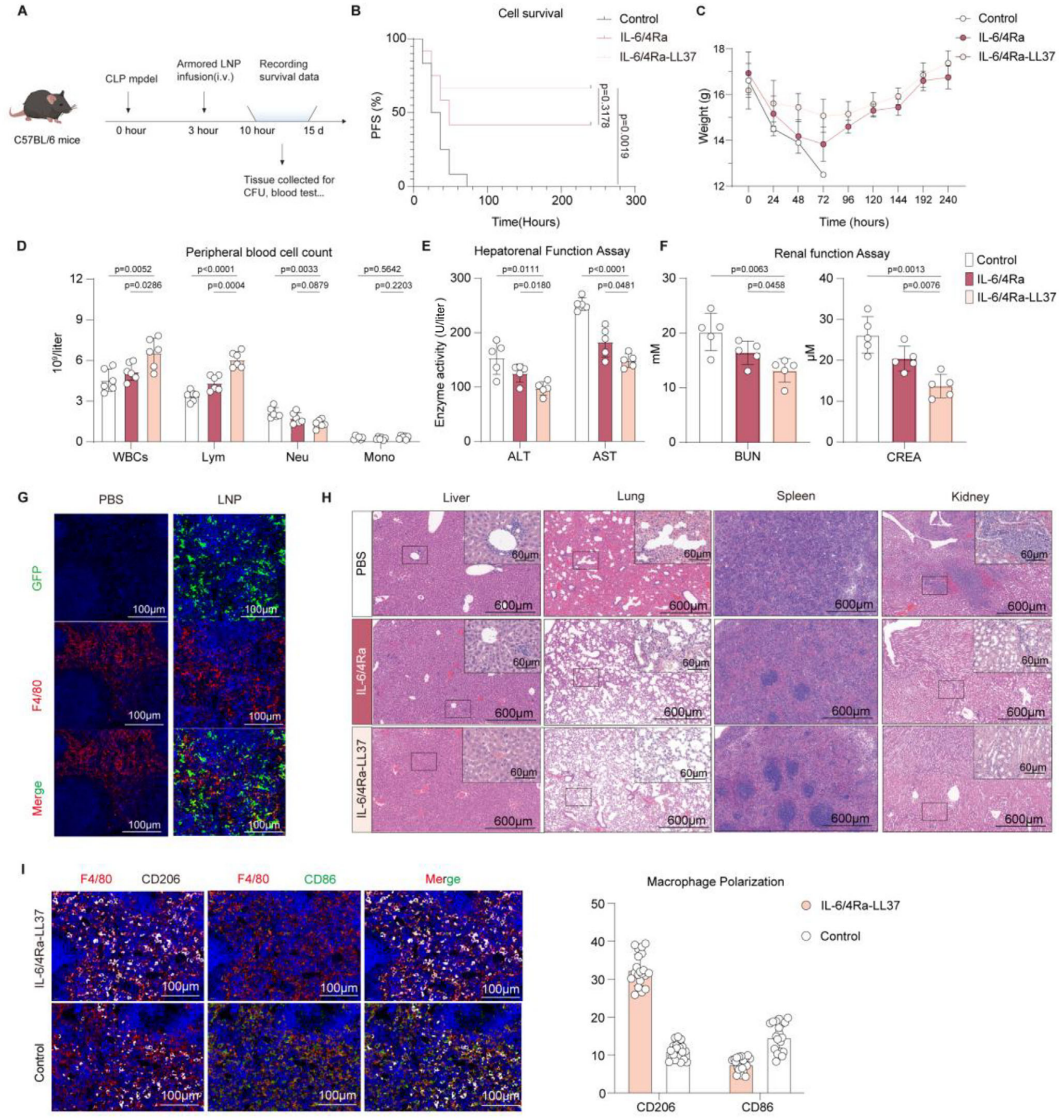
In vivo therapeutic efficacy of LL37‐fused engineered macrophages in mouse CLP model. (A) Schematic of the CLP model and F4/80‐targeted LNP‐mediated mRNA delivery. (B) Kaplan–Meier survival analysis (*n* = 12). (C) Body‐weight changes over time (*n* = 6). (D) Peripheral blood cellular composition, including WBCs, Lym, Neu, and Mono (*n* = 6). (E,F) Hepatic and renal functional recovery assessed by ALT/AST and BUN/CREA, respectively (*n* = 5). (G) In vivo expression of IL‐6/4Ra–GFP and colocalization with F4/80‐positive macrophages in the spleen. (H) H&E histopathology of major organs (liver, lung, spleen, and kidney). (I) Immunofluorescence and quantification of macrophage polarization markers (CD206 and CD86).

Survival analysis demonstrated that, compared with the control group, IL‐6/4Ra treatment provided a partial improvement in survival in CLP mice, whereas IL‐6/4Ra‐LL37 treatment resulted in a marked increase in survival, indicating a stronger protective effect against sepsis (Figure 7B). Consistently, dynamic body‐weight monitoring showed that control mice experienced pronounced weight loss after CLP, with delayed recovery in the early phase. In contrast, mice treated with IL‐6/4Ra‐LL37 exhibited less weight loss and a faster recovery trend over the subsequent observation period, suggesting that this therapeutic strategy effectively ameliorates the overall disease status induced by CLP (Figure 7C).

We next examined peripheral blood cellular composition. CLP induction significantly altered peripheral leukocyte and lymphocyte levels. Treatment with IL‐6/4Ra‐LL37 significantly restored peripheral immune cell counts, particularly recovering leukocyte and lymphocyte numbers, without causing abnormal increases in monocytes. These results suggest that the proposed therapeutic approach helps correct sepsis‐associated immune cell dysregulation (Figure 7D). In addition, liver and kidney function assays revealed substantial multi organ dysfunction in CLP mice, as reflected by elevated ALT, AST, BUN, and CREA. Following IL‐6/4Ra‐LL37 treatment, these hepatic and renal injury indicators were improved to varying degrees. Collectively, these findings indicate that IL‐6/4Ra‐LL37 engineered macrophage therapy alleviates CLP‐induced liver and kidney functional damage, demonstrating a favorable organ‐protective effect (Figure 7E,F).

To verify the ability of F4/80‐targeted LNPs to mediate in vivo expression of IL‐6/4Ra, we constructed an IL‐6/4Ra‐GFP fusion reporter system. Specifically, GFP was fused to the C‐terminus of IL‐6/4Ra via a flexible G4S linker to enable visualization of in vivo IL‐6/4Ra expression. Fluorescence imaging was then performed to assess expression in the spleen. As shown, compared with the PBS control, LNP treatment led to a clear GFP fluorescence signal in the spleen and displayed good spatial overlap with F4/80‐positive macrophage regions, indicating that F4/80‐targeted LNPs can effectively mediate the in situ expression of IL‐6/4Ra in splenic macrophages (Figure 7G). To quantitatively evaluate in vivo macrophage targeting efficiency, peritoneal lavage cells were analyzed by flow cytometry after LNP administration. The proportion of GFP^+^F4/80^+^ macrophages was markedly increased in the IL‐6/4Ra‐GFP group (25.4%) compared with PBS controls (1.09%), indicating robust macrophage specific delivery and expression (Extended Data Figure S13). This result provides direct in vivo evidence for therapeutic expression, supporting the subsequent evaluation of treatment efficacy.

Consistent with the above functional improvements, histopathological analyses further confirmed that the therapeutic system confers protection against CLP induced multi organ injury. H&E staining showed that the control group exhibited varying degrees of inflammatory cell infiltration, disrupted tissue architecture, and substantial tissue damage in the liver, lung, spleen, and kidney. IL‐6/4Ra treatment partially alleviated these pathological changes, while IL‐6/4Ra‐LL37 treatment preserved tissue architecture more effectively, with reduced inflammatory infiltration and markedly attenuated tissue injury across major organs (Figure 7H). These observations suggest that LL37 fusion enhances the tissue‐protective capacity of engineered macrophages in the CLP sepsis model.

Furthermore, we assessed macrophage phenotypic changes after IL‐6/4Ra treatment by immunofluorescence. Compared with the control group, IL‐6/4Ra treatment markedly increased CD206 fluorescence signal in F4/80 positive macrophages, while CD86 fluorescence signal decreased. These results indicate that treatment promotes a shift from a pro‐inflammatory M1‐like phenotype toward an anti‐inflammatory, reparative M2‐like phenotype (Figure 7I). Together, these findings demonstrate that IL‐6/4Ra can effectively reprogram macrophages in the CLP model, and that LL37 fusion further provides additional antibacterial activity and organ‐protective advantages.

Taken together, the CLP model results further demonstrate that F4/80‐targeted LNPs delivering IL‐6/4Ra‐LL37 engineered macrophages not only enhance survival, facilitate body weight recovery, and restore peripheral immune homeostasis, but also mitigate liver and kidney dysfunction as well as multi‐organ pathological damage. Mechanistically, this strategy achieves therapeutic benefits through concurrent induction of macrophage M2 polarization, inflammatory regulation, and enhanced antibacterial functionality, thereby showing substantial therapeutic potential in the clinically relevant CLP sepsis model.

## 4 Discussion

Our study demonstrates that engineered macrophages expressing IL6/4Ra fusion protein and LL37 antimicrobial peptide significantly improve survival outcomes in murine bacterial sepsis while simultaneously addressing multiple pathophysiological dimensions that conventional therapies fail to target. The increased survival rate and substantial reduction in serum IL‐6 levels validate our core hypothesis that IL‐6‐responsive immune modulation can effectively interrupt sepsis‐associated cytokine storms. More significantly, our platform achieved inflammatory control without compromising antimicrobial defense—bacterial burden in the organ decreased as well. This dual achievement directly addresses a fundamental limitation of previous immunomodulatory approaches: the CORTICUS trial's failure with hydrocortisone [35] and increased infection rates observed with anti‐TNF‐α therapies [36] both reflect the inherent risk of indiscriminate immunosuppression. Our platform circumvents this trade‐off through integrated design features that couple anti‐inflammatory activity spatiotemporally to sites of excessive cytokine production while maintaining constitutive antimicrobial capacity. Perhaps most clinically relevant [37, 38], engineered macrophage therapy substantially reduced T cell exhaustion markers (PD‐1, TIM‐3 expression decreased significantly) in surviving animals, suggesting potential for improving not only acute survival but post‐sepsis immunological recovery, a critical clinical gap given that sepsis survivors face a 5.68‐fold increased mortality from secondary infections within one year post‐discharge [39].

Our engineered macrophage platform addresses these multidimensional challenges through integrated design innovations. Incorporating IL‐6 receptor enables active cytokine capture from inflammatory microenvironments, functioning as a molecular “sink” that simultaneously reduces local cytokine concentration and initiates therapeutic signal transduction—a dual functionality absent in passive receptor systems. In our system, surface‐expressed IL‐6 receptor not only depletes pathological IL‐6 concentrations (which can exceed 1000 pg/mL in septic patients [37]) but simultaneously converts this inflammatory signal into therapeutic M2 polarization through engineered IL‐4Ra coupling. For instance, systemic IL‐4 administration or constitutive IL‐4 expression would drive global M2 polarization, potentially impairing pathogen clearance in non‐inflamed organs and increasing susceptibility to opportunistic infections [37]. Our design exploits the elevated IL‐6 environment characteristic of sepsis to drive localized M2 polarization. In vitro studies demonstrated that IL‐6 stimulation dose‐dependently induced M2 marker expression (CD206, Arginase‐1) in macrophages. In septic models characterized by elevated IL‐6 levels, engineered macrophages isolated from the spleen showed enhanced M2 phenotype markers. This observation supports the capacity of IL‐6 to promote M2 polarization of engineered macrophages in vivo. Strategic LL37 antimicrobial peptide expression counterbalances anti‐inflammatory polarization risks by maintaining broad‐spectrum bactericidal activity. LL37, a 37 amino acid human cathelicidin, exhibits potent activity against MDR pathogens through membrane disruption mechanisms that circumvent conventional resistance pathways. Critically, LL37 demonstrates efficacy against methicillin‐resistant *S. aureus* (MRSA), vancomycin‐resistant Enterococci (VRE), and carbapenem‐resistant Klebsiella pneumoniae, WHO critical‐priority pathogens that account for a substantial proportion of sepsis‐associated mortality in immunocompromised patients [40, 41]. For example, studies have demonstrated that LL‐37 effectively eradicates MRSA biofilms at sub‐MIC concentrations, displaying superior antibiofilm activity compared to conventional antibiotics such as vancomycin and gentamicin, even at concentrations 100‐fold lower [42]. This integrated design—coupling context‐dependent immunomodulation with constitutive antimicrobial capacity maintains host defense integrity even during therapeutic immune suppression phases, thereby addressing a critical vulnerability of anti‐inflammatory monotherapies. Our comparative analysis revealed the necessity of this integration: IL‐6 antibody monotherapy reduced systemic inflammation but failed to control bacterial proliferation, resulting in less effective survival benefit.

Our in vivo efficacy studies revealed that engineered macrophage therapy not only significantly improved 7‐day survival but also substantially reduced T cell exhaustion markers (PD‐1, TIM‐3) in surviving animals. This finding carries profound clinical implications: attenuated T cell exhaustion likely preserves adaptive immune memory formation and maintains immunocompetence against secondary infections, a major cause of delayed mortality in sepsis survivors [43, 44]. By simultaneously controlling acute hyperinflammation while preventing downstream immune paralysis, our platform addresses both phases of sepsis‐associated immune dysregulation, an achievement unattained by current single‐target therapies. Flow cytometric analysis at day 3 post‐infection revealed that IL6/4Ra‐LL37 treated animals exhibited significantly lower PD‐1^+^ and TIM‐3^+^ CD8^+^ T cells compared to untreated survivors. This mechanistic insight extends therapeutic objectives beyond immediate crisis management to encompass long‐term immunological recovery, potentially improving post‐discharge outcomes that remain a major clinical challenge. The preservation of T cell function may also have implications for vaccination responses and cancer surveillance in sepsis survivors, domains increasingly recognized as compromised in this population [45].

The implementation of F4/80‐targeted lipid nanoparticle (LNP)‐mediated in situ transfection represents a transformative advancement in cellular immunotherapy logistics. Traditional ex vivo cell manufacturing protocols requiring peripheral blood harvest, cell isolation, ex vivo culture, lentiviral transduction, expansion (typically 7–14 days), quality control testing [45], and cryopreservation, impose prohibitive time constraints incompatible with acute sepsis management, where therapeutic intervention must occur within hours of diagnosis to prevent irreversible organ damage. For example, FDA‐approved CAR‐T therapies require 3–4 weeks from leukapheresis to product availability, an unacceptable timeline for septic patients with hourly mortality risk escalation [46]. Our in situ engineering approach circumvents these logistical barriers by directly converting endogenous tissue‐resident macrophages into therapeutic effectors within 6 h post‐administration through a single intravenous injection, enabling clinically feasible implementation in emergency settings. Recent advances in macrophage‐targeted LNP technology have demonstrated higher transfection efficiency in liver and spleen using F4/80 antibody conjugation or mannose receptor targeting [47, 48], validating the translational potential of this platform. This paradigm shift, from pre‐manufactured product models to on‐demand, in situ generation which fundamentally reconfigures cellular immunotherapy applicability for acute critical illness.

Despite these advances, key limitations warrant discussion. First, our intraperitoneal *S. aureus* model provides a useful reductionist framework but does not fully capture the clinical heterogeneity of sepsis. To address this limitation, we further validated the therapeutic efficacy in a cecal ligation and puncture (CLP) model, which induces polymicrobial sepsis and better recapitulates the inflammatory and immune dysregulation observed in patients, thereby extending the translational relevance beyond a single pathogen–driven setting. Future validation should still include additional clinically relevant models, such as pneumonia‐induced and fungal sepsis [49].

Second, while achieving higher transfection in spleen/liver, F4/80‐LNPs showed lower efficiency in lung and kidney, critical organs driving ARDS and AKI mortality [50, 51]. This reflects LNP biodistribution favoring reticuloendothelial organs and off‐target transfection in non‐macrophage cells [52, 53], raising concerns about cell type specific cytokine signaling effects. Future strategies should incorporate some complementary approaches to address these delivery limitations. Critically, we did not assess survival against secondary infections; future studies must validate whether preserved T cell function provides durable immunocompetence using sequential infection models.

These findings establish engineered macrophages as an innovative sepsis therapeutic platform integrating complementary mechanisms: IL‐6 capture, conditional M2 polarization, LL37‐mediated bacterial killing, T cell exhaustion prevention, and rapid in situ generation. This approach transcends single‐target strategies that have failed clinically. Despite limitations in disease model diversity and organ‐specific delivery efficiency, this concept demonstrates multidimensional efficacy spanning survival, bacterial clearance, and immune preservation. Future integration of patient specific cytokine profiling, advanced targeting vectors, and combination therapeutics promises clinical transformation. The platform's modular design provides a generalizable framework for engineered cellular immunotherapy in critical illness.

## 5 Conclusions

This study reports an innovative in situ LNP‐mediated multifunctional engineered macrophage therapeutic system for sepsis treatment, demonstrating substantial therapeutic efficacy through integrated immune modulation and antimicrobial capacity (Figure 8). First, we successfully developed an F4/80‐targeted lipid nanoparticle platform achieving macrophage‐selective transgene delivery with high specificity. Second, we constructed a chimeric IL6/4Ra receptor by rational fusion of the IL‐6 receptor extracellular domain with the IL‐4 receptor intracellular domain, simultaneously achieving dual critical functions: selective IL‐6 capture and sequestration paired with M2 macrophage polarization promotion. To overcome potential antimicrobial deficiency associated with M2 polarization promotion, we incorporated the LL37 antimicrobial peptide, conferring potent broad spectrum antimicrobial capacity. Critically, LL37 fusion did not compromise M2 polarization promoting function, maintaining elevated M2 marker gene expression while effectively reducing bacterial colony forming units. Clinical translational potential was comprehensively validated in human derived macrophages. We tested LNP‐F4/80‐mediated in situ engineering of IL6/4Ra‐LL37‐expressing macrophages, which achieved simultaneous accomplishment of four critical therapeutic objectives in *S. aureus* sepsis mouse models: substantially improved survival rates, significantly reduced multi organ bacterial burden across liver, spleen, kidney, and lung, effective mitigation of sepsis induced organ dysfunction and inflammatory injury, and profound restoration of T cell function. Collectively, these findings demonstrate that engineered macrophages successfully integrate inflammatory control, antimicrobial action, and immune function restoration within a single cellular platform. This innovative strategy represents a paradigm shift in sepsis immunotherapy, establishing engineered macrophages as a conceptually superior and mechanistically integrated therapeutic approach for precision treatment of sepsis associated biphasic immune dysregulation.

**FIGURE 8:**
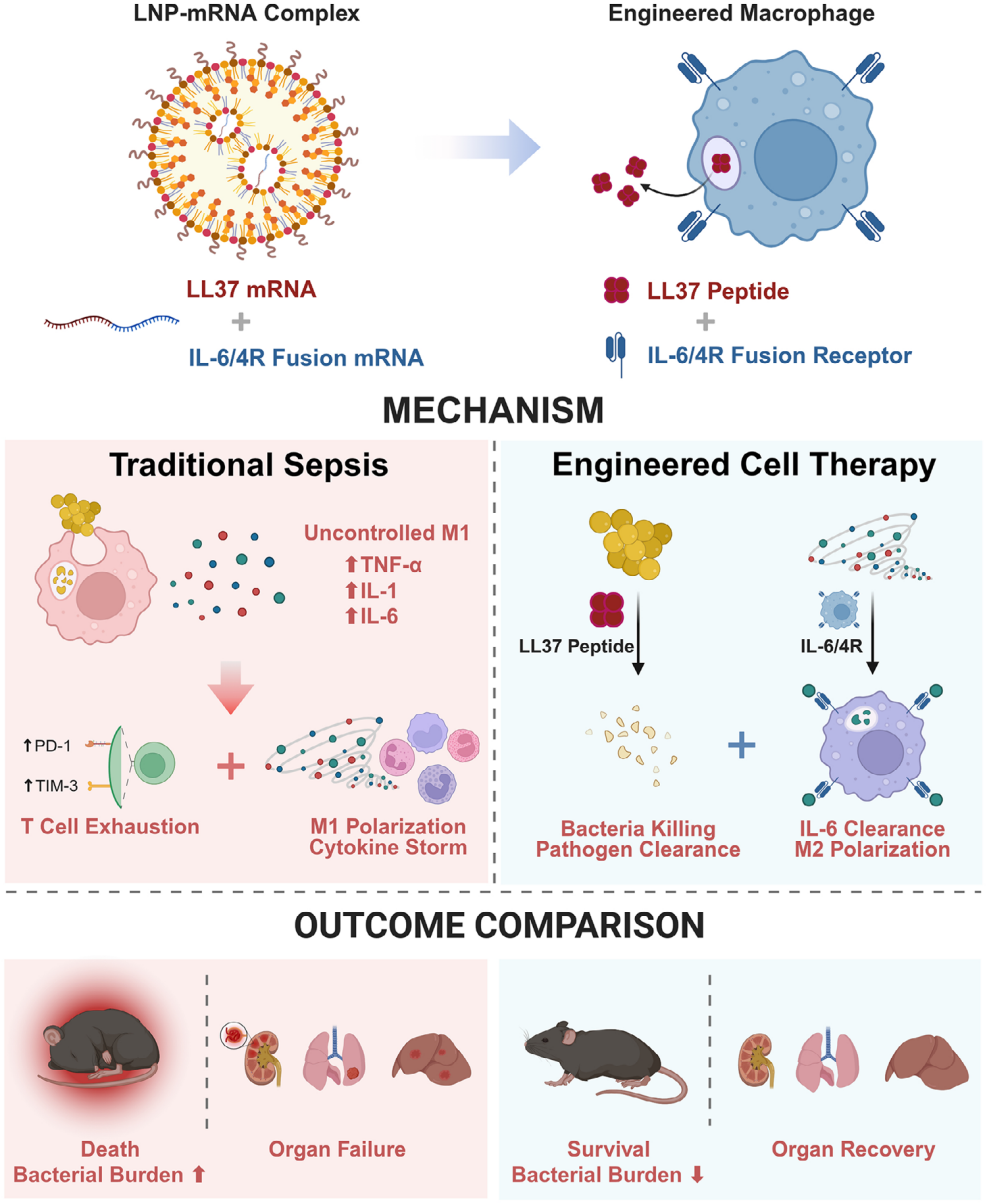
LNP‐mRNA engineered macrophages combat sepsis via dual mechanisms of bacterial clearance and cytokine storm suppression.

## Author Contributions

**Tianyang Jie**: Writing – original draft, Methodology, Investigation, Data curation, Conceptualization. **Zheyu Zhang**: Writing – original draft, Software, Data curation. **Hao Zhang**: Formal analysis, Methodology, Investigation, Data curation. **Linke Bian**: Writing – original draft, Methodology, Investigation. **Penglong Zheng**: Writing – original draft. **Sijia Zhou**: Software, Formal analysis, Data curation. **Minqi Wang**: Formal analysis, Data curation. **Mingyu Qu**: Methodology, Investigation. **Yufei Wang**: Formal analysis, Data curation. **Xinhua Qu**: Writing – review & editing, Writing – original draft, Validation, Supervision, Funding acquisition, Conceptualization. **Bing Yue**: Writing – review & editing, Writing – original draft, Validation, Supervision, Funding acquisition, Conceptualization

## Ethics Approval

All animal experiments adhered to the animal life protection standards and procedures sanctioned by Shanghai Rat & Mouse Biotech Co., Ltd (Issue No. IACUC‐2025‐1‐024). This study does not involve human subjects.

## Conflicts of Interest

The authors declare no conflicts of interest.

## Supporting information




**Supporting File**: advs77016‐sup‐0001‐SuppMat.rtf.

## Data Availability

The data that support the findings of this study are available from the corresponding author upon reasonable request.
