# Supplementary material for: Chimeric IL‐6/4R‐LL37 Engineered Macrophages Achieve Synchronized Inflammation Control and Antimicrobial Defence in Sepsis
Source: Adv Sci (Weinh). 2026 Aug 7;13(60):e77016. doi: 10.1002/advs.77016 (PMC13449531; doi:10.1002/advs.77016)
Supplement: Supplementary file 1 [file ADVS-13-e77016-s001.rtf]

Appendix A. Supplementary data
The following is the Supplementary data to this article:

Extended Figure. 1 Body weight changes in mice following IL-6 antibody treatment

Extended Figure. 2 Flow cytometric validation of bone marrow-derived macrophage (BMDM) differentiation


Extended Figure. 3. Physicochemical characterization of the LNP formulation. 


Extended Figure. 4 Cell viability assessment of LNP-GFP treated macrophages at multiple time points


Extended Figure. 5 In vivo biosafety evaluation of LNP assessed by hepatic and renal function markers (ALT, AST, BUN, and CREA)

Extended Figure. 6 Histological analysis of major organs by hematoxylin and eosin (H&E) staining following LNP transfection


Extended Figure. 7 Functional validation of IL6/4Ra in human-derived macrophages.


Extended Figure. 8 (A) IL-6/4R expression attenuates inflammatory cytokine production BMDMs. (B) IL-6/4Ra expression promotes M2-associated mRNA expression in human peripheral blood-derived macrophages


Extended Figure. 9 Phenotypic changes of human IL-6/4Ra-LL37 engineered macrophages co-cultured with S. aureus


Extended Figure. 10 Gating strategy for peripheral blood neutrophils

Extended Figure. 11 Gating strategy for spleen T cells

Extended Figure. 12 Gating strategy for spleen Macrophages

Extended Figure. 13 GFP⁺F4/80⁺ macrophages in peritoneal lavage cells


Tables
Tables S1
Gene 	Forward (5'-3')	Reverse (5'-3')	
Arginase (M)	agtctggcagttggaagcat	ctggttgtcaggggagtgtt	
FIZZ1 (M)	tgctgggatgactgctactg	ctgggttctccacctcttca	
IL-10 (M)	ccagtacagccgggaagaca	cagctggtcctttgtttgaaaga	
CD209(H)	aatggctggaacgacgacaaa	caggaggctgcggacttttt	
CCL22(H)	cctactctgatgaccgtggc	agggaatgcagagagttggc
